# Exploring Research Trends in Chronic Kidney Disease and Sedentary Behavior: A Text Mining Analysis of Article Titles

**DOI:** 10.7759/cureus.109944

**Published:** 2026-05-30

**Authors:** Akihiro Yokoyama, Nobuhiro Nasu, Akihiko Katayama, Shuhei Hishii, Keiichi Namio, Kentaro Sugahara, Hiroaki Kataoka, Hiromi Suzuki, Nobuyuki Miyatake

**Affiliations:** 1 Department of Hygiene, Faculty of Medicine, Kagawa University, Kagawa, JPN; 2 Department of Physical Therapy, Okayama Healthcare Professional University, Okayama, JPN; 3 Faculty of Social Sciences, Shikoku Gakuin University, Kagawa, JPN; 4 Department of Human Sciences, Major of Physical Therapy, Kibi International University, Takahashi, JPN

**Keywords:** article titles, chronic kidney disease, correspondence analysis, sedentary behavior, text mining analysis

## Abstract

Sedentary behavior (SB) has recently gained attention across various diseases due to its relationship with disease onset, quality of life, and prognosis. In the present study, we searched for journal article titles associated with the relationship between chronic kidney disease (CKD) and SB and used an exploratory text mining analysis to conduct an exploratory examination of research trends.

On January 1, 2026, we entered “chronic kidney disease and sedentary behavior” into the PubMed^®^ article search engine and downloaded article titles, authors, journal names, and publication years. Using the text mining software KH Coder^® ^(KH Coder 3.0, Koichi Higuchi, Japan), we extracted frequently occurring words from article titles and performed a correspondence analysis by time period (up to 2015, 2016-2020, and 2021-2025) and survey location (Japan and outside Japan). The search results found 178 articles. The total number of words obtained for article titles was 2,890. The top five most frequent words were “kidney” (91 words), “disease” (88 words), “physical” (78 words), “activity” (68 words), and “chronic” (68 words). In the correspondence analysis by time period, “randomize”, “intervention”, and “cohort” were characteristic. “Randomize” was characteristic from 2021 to 2025. In the analysis by survey location, only eight out of all articles conducted surveys in Japan, indicating that surveys in Japan were fewer, with most originating from outside Japan.

Research investigating the relationship between CKD and SB has recently begun to see the implementation of high-level evidence studies, such as cohort studies and randomized controlled trials. Furthermore, many of these studies appear to have been conducted outside Japan.

## Introduction and background

Patients with chronic kidney disease (CKD) are generally elderly, often have complications, and also have low levels of physical activity [1-3]. The latter characteristic may be described as not only “inactive”, but also “excessively sedentary” [2-8].

Sedentary behavior (SB) has recently gained attention as a health risk factor independent of physical activity levels [4-12]. It has also been associated with the onset of various diseases, quality of life (QOL), and prognosis [9-12]. Henson et al. reported that SB was related to an increased prevalence of multiple chronic diseases [9]. Xavier et al. also demonstrated that increased SB resulted in a decline in QOL among patients with Chagas disease [10]. Furthermore, a systematic review by de Rezende et al. showed that increased SB was associated with mortality, fatal and nonfatal cardiovascular diseases, type 2 diabetes, metabolic syndrome, waist circumference, and being overweight/obese [11,12].

We previously investigated the relationship between SB and QOL in patients with chronic hemodialysis (CHD) using cross-sectional and longitudinal studies [4-8]. Hishii et al., Namio et al., and Sugahara et al. reported correlations between SB and prolonged sedentary bouts, and between EuroQol 5-Dimensions 3-level scores, in CHD patients, suggesting a relationship with health-related QOL in cross-sectional studies [4-6]. In addition, Hishii et al. described the relationship between SB and all-cause mortality in patients with CHD using a longitudinal study [7]. Namio et al. also showed that prolonged sedentary bouts were closely associated with all-cause mortality in a prospective cohort study [8].

Text mining is a research method for qualitatively evaluating text and audio data. In recent years, it has been increasingly used in medical research fields [13-19]. Conceição and Couto introduced text mining research in the field of cancer [13]. We also used text mining analysis in studies investigating impressions of COVID-19 vaccines, human papillomavirus vaccines, AIDS, and syphilis [14-16]. In recent years, the use of text mining analysis has increased to efficiently extract information from large volumes of medical literature data [17-19].

However, studies have yet to examine the literature on the relationship between CKD and SB using text mining analysis. Since research on CKD and SB is insufficient, it is important to conduct an exploratory analysis of existing studies in order to identify areas for future research. The titles given to academic papers often convey their content efficiently and concisely. Therefore, the present study aimed to collect article titles related to CKD and SB and to clarify research trends through an exploratory text mining analysis.

## Review

Methods

On January 1, 2026, we entered “chronic kidney disease and sedentary behavior” into the PubMed® article search engine and downloaded PubMed ID, Title, Authors, Citation, First Author, Journal/Book, Publication Year, Create Date, PubMed Central ID, NIH Manuscript Submission System ID, and DOI. In the search's custom filter, the publication date was set to 2026 or earlier; “Text availability”, “Article attribute”, and “Article type” were left blank; “All results” was selected under “Save citations to file”; the format was set to CSV; and the data file was downloaded. As a preprocessing step, all uppercase letters in article titles were converted to lowercase. Furthermore, issuance years were categorized into three groups: “Up to 2015”, “2016-2020”, and “2021-2025”. Survey locations were also classified by verifying only the authors’ names and distinguishing between Japan and outside Japan. Article titles were then analyzed using the text mining software KH Coder® (KH Coder 3.0, Koichi Higuchi, Japan) [20,21]. Words were automatically extracted from the article titles collected by PubMed® using KH Coder® to create a list of the frequency of their occurrence. KH Coder® was performed with default settings to eliminate the bias of the analyst’s opinion as much as possible, and default settings were also used for the morphological analysis dictionary, which used the default Stanford Part-of-Speech Tagger, and no settings were configured for “force pick up” or “force ignore” terms. Furthermore, compound words were treated as separate words rather than a single word. We then performed a correspondence analysis [20,21] to visualize the relationships between words in a scatter plot. Classification criteria were the time period (“up to 2015”, “2016-2020”, and “2021-2025”) and survey location (“Japan” and “outside Japan”). The settings for the correspondence analysis were configured with a minimum frequency of five, a minimum number of documents of one, and the top 60 words. Additionally, in the word display for the correspondence analysis, “ckd” was converted into “CKD” and “nhanes” to “NHANES” and displayed in uppercase.

Results

The search results found 178 articles. All downloaded data are included in the Appendix. The total number of words obtained for article titles was 2,890. The top 10 most frequent words were “kidney” (91 words), “disease” (88 words), “physical” (78 words), “activity” (68 words), “chronic” (68 words), “sedentary” (52 words), “study” (44 words), “patient” (41 words), “adult” (33 words), and “behavior” (32 words). The top 20 most frequently occurring words are indicated in Table 1.

**Table 1:** Frequently occurring words (totally 2,890 words)

S.no.	Word	Number of words	Percentage
1	Kidney	91	3.15
2	Disease	88	3.04
3	Physical	78	2.70
4	Activity	68	2.35
5	Chronic	68	2.35
6	Sedentary	52	1.80
7	Study	44	1.52
8	Patient	41	1.42
9	Adult	33	1.14
10	Behavior	32	1.11
11	Risk	25	0.87
12	Association	24	0.83
13	Function	19	0.66
14	Factor	18	0.62
15	Older	18	0.62
16	Time	18	0.62
17	Cohort	16	0.55
18	Renal	16	0.55
19	Exercise	15	0.52
20	Health	15	0.52

The number of reports by time period was as follows: “up to 2015” (Japan: 0 reports, outside Japan: 47 reports), “2016-2020” (Japan: one report, outside Japan: 43 reports), and “2021-2025” (Japan: seven reports, outside Japan: 80 reports) (Table 2).

**Table 2:** Number of reports from Japan and outside Japan by time period

Time period	Japan	Outside Japan	Total
Up to 2015	0	47	47
2016-2020	1	43	44
2021-2025	7	80	87
Total	8	170	178

**Figure 1:**
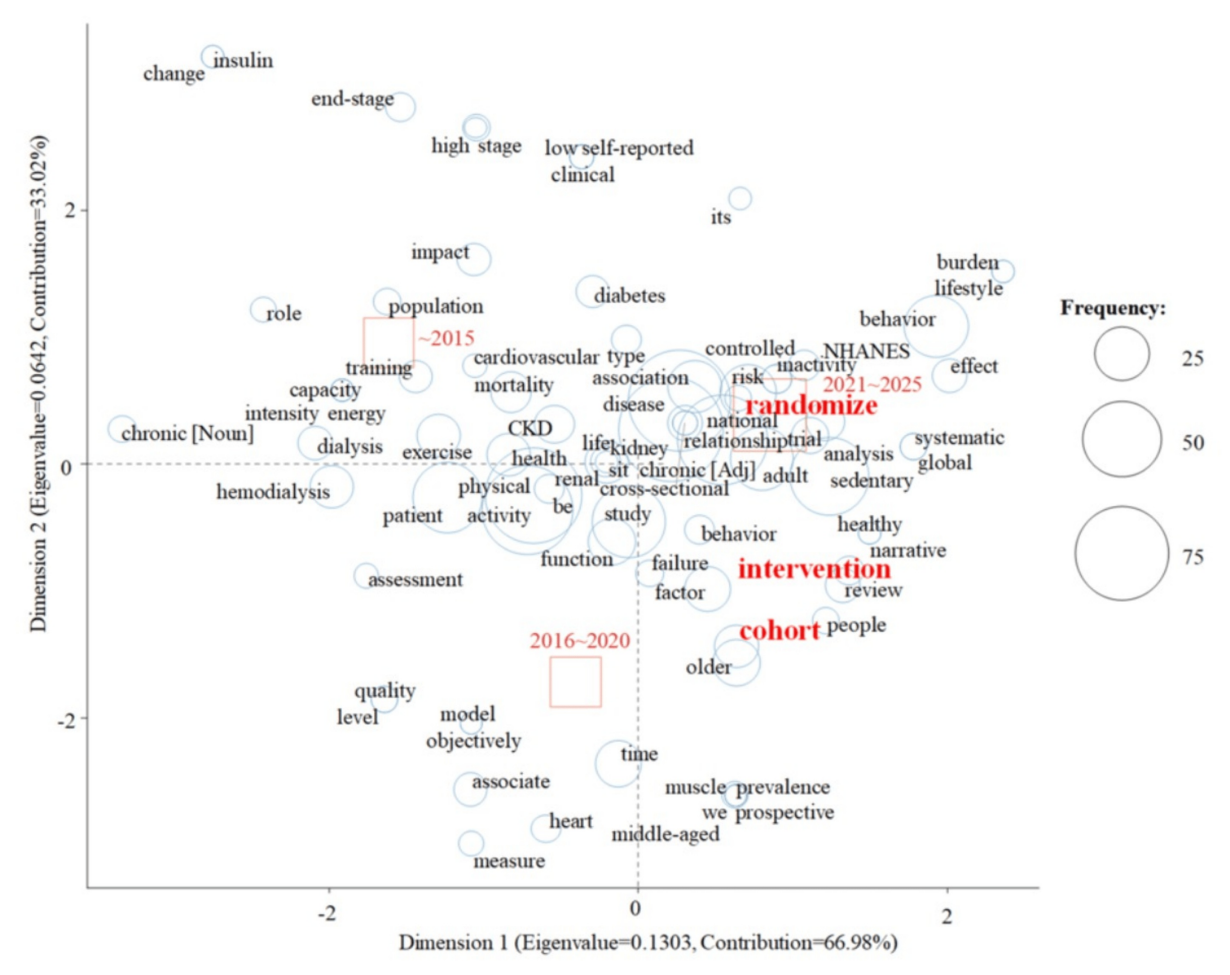
Correspondence analysis by time period The origin is the point where the dotted lines connecting the 0-point on the vertical axis and the 0-point on the horizontal axis intersect. Terms closer to the origin have higher centrality (the strength of their co-occurrence relationships with other terms) Image credit: This is an original image created by the author Akihiro Yokoyama using KH Coder (Koichi Higuchi, Tokyo, Japan)

In the correspondence analysis by time period, “randomize” was characteristic of the “2021-2025” period. “Intervention” and “cohort” were recognized in the intermediate between the “2016-2020” and “2021-2025” classifications. “Cross-sectional” and “study” were positioned in the middle of the three groups: “up to 2015”, “2016-2020”, and “2021-2025”. Furthermore, “chronic”, “kidney”, “disease”, and “CKD” were similarly positioned in the middle of the three groups. The results of the correspondence analysis by time period are indicated in Figure 1. Component 1 on the horizontal axis had an eigenvalue of 0.1303 and a contribution of 66.98%, while Component 2 on the vertical axis had an eigenvalue of 0.0642 and a contribution of 33.02%. Terms closer to the origin have higher centrality (the strength of their co-occurrence relationships with other terms). The origin is the point where the dotted lines connecting the 0-point on the vertical axis and the 0-point on the horizontal axis intersect.

**Figure 2:**
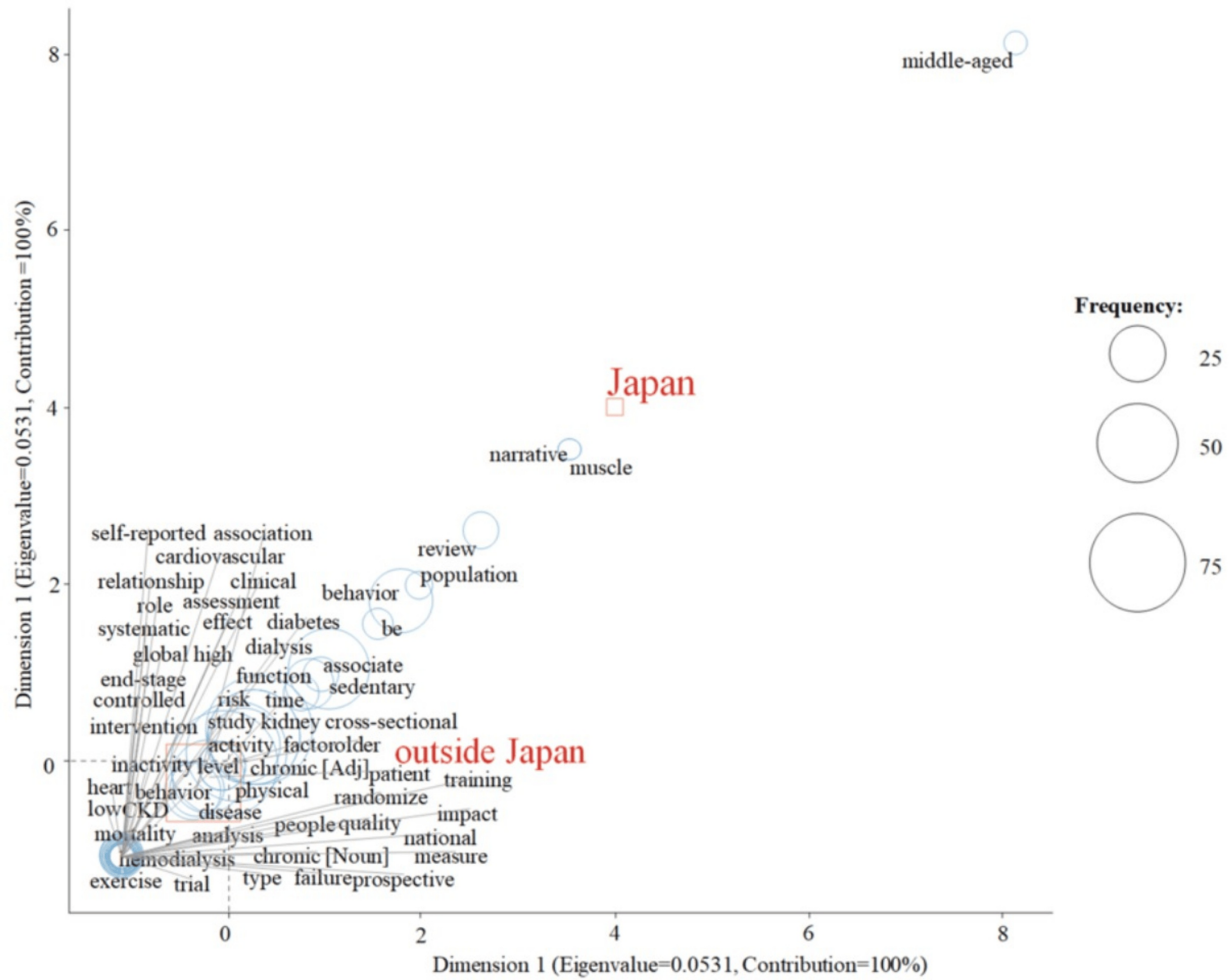
Correspondence analysis by survey location The origin is the point where the dotted lines connecting the 0-point on the vertical axis and the 0-point on the horizontal axis intersect. Terms closer to the origin have higher centrality (the strength of their co-occurrence relationships with other terms) Image credit: This is an original image created by the author Akihiro Yokoyama using KH Coder (Koichi Higuchi, Tokyo, Japan)

Furthermore, in the analysis by survey location, eight reports were conducted in Japan, while the remaining 170 originated from outside Japan. The results of the correspondence analysis by survey location are shown in Figure 2. In Figure 2, both the horizontal and vertical axes show an eigenvalue of 0.0531 and a contribution rate of 100%. Terms closer to the origin have higher centrality (the strength of their co-occurrence relationships with other terms). The origin is the point where the dotted lines connecting the 0-point on the vertical axis and the 0-point on the horizontal axis intersect.

Discussion

In the present study, we entered “chronic kidney disease and sedentary behavior” into PubMed® and performed a text mining analysis of the downloaded article titles. The search results found 178 articles.

In the correspondence analysis focusing on the terms “randomize”, “intervention”, and “cohort”, the word “randomize” was characteristic of the time period “2021-2025”. In the analysis by survey location, only eight of all articles conducted surveys in Japan, indicating that surveys in Japan were clearly fewer, with most reports originating from outside Japan.

In recent years, the use of text mining analysis has increased to efficiently extract information from large volumes of medical literature data [17-19]. Forsgren et al. described programming methods that use text mining analysis to assist with screening and data extraction in systematic reviews [17]. Wang and Lo also proposed a method for visualizing and summarizing literature on COVID-19 [18]. Furthermore, Fukushima et al. analyzed and reported on literature regarding peritoneal dialysis published in PubMed® using artificial intelligence text mining [19].

In the present study, text mining analysis of journal article titles related to CKD and SB revealed that high-level evidence studies, including cohort studies and randomized controlled trials (RCTs), have been conducted only in recent years. This may be attributed to increased interest in lifestyle behaviors resulting from the aging of CKD patients and their longer survival [1]. Moreover, factors include the international recognition of SB as a health risk distinct from physical inactivity [4-7] and assessments of SB becoming more straightforward due to the widespread use of accelerometers and wearable devices [2,3,8-12].

In the present study, 178 articles were found. Since this is not a particularly large number, research on CKD and SB is still in its early stages. Furthermore, limited research has been conducted in Japan. One possible reason for this is that CKD research in Japan has generally focused on laboratory values, prognosis, and complications, with a minimal emphasis on behavioral epidemiology. Furthermore, the concept of SB itself is relatively new in Japan, which may have contributed to the delay in its adoption in research. In addition, there is a lack of large-scale behavioral data on CKD patients in the conservative management phase, and there is a need to accumulate more research data in Japan in the future.

Limitations

This study does not suggest a causal relationship between CKD and SB. Moreover, this study is not intended to demonstrate the effectiveness of intervention strategies; rather, it is a preliminary study aimed at identifying trends in the field through a text mining analysis and identifying priority areas for future research. Therefore, this study has several limitations that need to be addressed. This study was based on an analysis of titles; since the full text of each article was not read, there are limits to how well the details of their content were grasped. Furthermore, the choice of terminology may have had an impact. The term “sedentary behavior” was selected for this study, which itself may have introduced a selection bias in the search results. Another limitation is that the search engine used was limited to PubMed®, and the scope was restricted to English-language literature. Therefore, it did not fully cover all medical literature, including that written in Japanese or other languages. The increasing appearance of terms related to randomized and interventional designs suggests a possible shift toward more rigorous study designs, although confirmation through abstract or full-text review is needed. Nevertheless, this method is extremely useful for gaining an understanding of research trends and general directions [19].

## Conclusions

We conducted an exploratory investigation of article titles on CKD and SB using text mining analysis. High-level evidence studies, such as cohort studies and RCTs, have been conducted on the relationship between CKD and SB in recent years, and many of these studies appear to have been performed outside Japan. Therefore, further accumulation of cohort studies and RCTs in Japan is needed in the future. These findings suggest that research on CKD and SB is still developing, and the field has not yet reached maturity. The observed patterns help clarify how research interests have shifted over time and highlight areas where further investigation is needed. Even though our analysis focused only on titles, it provides a useful overview of emerging themes and the current direction of the field. Continued efforts, especially within Japan, will be important for advancing understanding and improving patient care.

## Appendices

**Table 3:** Literature data downloaded from PubMed

PMID	Title	Authors	Citation	First author	Journal/book	Publication year	Create date	PMCID	NIHMS ID	DOI
34343989	Physical activity and health in chronic kidney disease	Wilund KR, Thompson S, Viana JL, Wang AY	Contrib Nephrol. 2021;199:43-55. doi: 10.1159/000517696. Epub 2021 Aug 3	Wilund KR	Contrib Nephrol	2021	2021/8/3	-	-	10.1159/000517696
40281378	Sedentary behavior and risk of chronic kidney disease: a systematic review and meta-analysis	Zhang L, Liu F, Li M, Fan Y	Int Urol Nephrol. 2025 Sep;57(9):2917-2926. doi: 10.1007/s11255-025-04536-9. Epub 2025 Apr 25	Zhang L	Int Urol Nephrol	2025	2025/4/25	-	-	10.1007/s11255-025-04536-9
36765338	Association between sedentary behavior and chronic kidney disease in Korean adults	Jang YS, Park YS, Kim H, Hurh K, Park EC, Jang SY	BMC Public Health. 2023 Feb 10;23(1):306. doi: 10.1186/s12889-022-14929-5	Jang YS	BMC Public Health	2023	2023/2/11	PMC9912677		10.1186/s12889-022-14929-5
33524598	Risk factors for early-onset colorectal cancer: a systematic review and meta-analysis	O'Sullivan DE, Sutherland RL, Town S, Chow K, Fan J, Forbes N, Heitman SJ, Hilsden RJ, Brenner DR	Clin Gastroenterol Hepatol. 2022 Jun;20(6):1229-1240.e5. doi: 10.1016/j.cgh.2021.01.037. Epub 2021 Jan 29	O'Sullivan DE	Clin Gastroenterol Hepatol	2022	2021/2/1	-	-	10.1016/j.cgh.2021.01.037
39026309	Lifestyle factors and their relative contributions to longitudinal progression of cardio-renal-metabolic multimorbidity: a prospective cohort study	Zhang N, Liu X, Wang L, Zhang Y, Xiang Y, Cai J, Xu H, Xiao X, Zhao X	Cardiovasc Diabetol. 2024 Jul 18;23(1):265. doi: 10.1186/s12933-024-02347-3	Zhang N	Cardiovasc Diabetol	2024	2024/7/18	PMC11264843		10.1186/s12933-024-02347-3
36894924	Association between sedentary behavior and depression in US adults with chronic kidney disease: NHANES 2007-2018	Liu L, Yan Y, Qiu J, Chen Q, Zhang Y, Liu Y, Zhong X, Liu Y, Tan R	BMC Psychiatry. 2023 Mar 9;23(1):148. doi: 10.1186/s12888-023-04622-1	Liu L	BMC Psychiatry	2023	2023/3/9	PMC9996893		10.1186/s12888-023-04622-1
39957059	Device-measured physical activity, sedentary behaviour and risk of chronic kidney diseases across levels of grip strength	He Y, Wang J, Zhang W, Chen X, Wu Q, Li Y, Ou Y, Liu Y, Feng H, Zhang J, Ai S, Liang YY, Ning Y, Zhang J	J Cachexia Sarcopenia Muscle. 2025 Feb;16(1):e13726. doi: 10.1002/jcsm.13726	He Y	J Cachexia Sarcopenia Muscle	2025	2025/2/17	PMC11830631		10.1002/jcsm.13726
37968170	Evaluating the effect of a digital health intervention to enhance physical activity in people with chronic kidney disease (Kidney BEAM): a multicentre, randomised controlled trial in the UK	Greenwood SA, Young HML, Briggs J, Castle EM, Walklin C, Haggis L, Balkin C, Asgari E, Bhandari S, Burton JO, Billany RE, Bishop NC, Bramham K, Campbell J, Chilcot J, Cooper NJ, Deelchand V, Graham-Brown MPM, Hamilton A, Jesky M, Kalra PA, Koufaki P, McCafferty K, Nixon AC, Noble H, Saynor Z, Taal MW, Tollit J, Wheeler DC, Wilkinson TJ, Worboys H, Macdonald JH	Lancet Digit Health. 2024 Jan;6(1):e23-e32. doi: 10.1016/S2589-7500(23)00204-2. Epub 2023 Nov 14	Greenwood SA	Lancet Digit Health	2024	2023/11/15	-	-	10.1016/S2589-7500(23)00204-2
40938524	Sedentary behavior as an emerging risk factor for chronic kidney disease: a narrative review	Yoshikoshi S, Kosaki K, Oka K, Maeda S, Yamagata K	Clin Exp Nephrol. 2025 Sep 12. doi: 10.1007/s10157-025-02764-y. Online ahead of print	Yoshikoshi S	Clin Exp Nephrol	2025	2025/9/12	-	-	10.1007/s10157-025-02764-y
35516443	Associations of sedentary time and physical activity with adverse health conditions: outcome-wide analyses using isotemporal substitution model	Cao Z, Xu C, Zhang P, Wang Y	EClinicalMedicine. 2022 Apr 28;48:101424. doi: 10.1016/j.eclinm.2022.101424. eCollection 2022 Jun	Cao Z	EClinicalMedicine	2022	2022/5/6	PMC9065298		10.1016/j.eclinm.2022.101424
40635243	Domain-specific physical activity and sedentary behavior in relation to chronic kidney disease: a cross-sectional analysis of 24,950 U.S. adults in NHANES 1999-2012	Zhang N, Shi J	Ren Fail. 2025 Dec;47(1):2525460. doi: 10.1080/0886022X.2025.2525460. Epub 2025 Jul 9	Zhang N	Ren Fail	2025	2025/7/10	PMC12247097		10.1080/0886022X.2025.2525460
40113344	The impact of leisure sedentary behaviors on risk of chronic kidney disease, diabetes, and related complications: Mendelian randomization study	Zhong S, Xiao R, Lin Y, Xie B, Sun J	Ren Fail. 2025 Dec;47(1):2479177. doi: 10.1080/0886022X.2025.2479177. Epub 2025 Mar 20	Zhong S	Ren Fail	2025	2025/3/20	PMC11926908		10.1080/0886022X.2025.2479177
25287433	Chronic kidney disease and premature ageing	Kooman JP, Kotanko P, Schols AM, Shiels PG, Stenvinkel P	Nat Rev Nephrol. 2014 Dec;10(12):732-42. doi: 10.1038/nrneph.2014.185. Epub 2014 Oct 7	Kooman JP	Nat Rev Nephrol	2014	2014/10/8	-	-	10.1038/nrneph.2014.185
32435869	Sedentary behavior and kidney function in adults: a narrative review	Volaklis K, Mamadjanov T, Meisinger C	Wien Klin Wochenschr. 2021 Feb;133(3-4):144-152. doi: 10.1007/s00508-020-01673-2. Epub 2020 May 20	Volaklis K	Wien Klin Wochenschr	2021	2020/5/22	-	-	10.1007/s00508-020-01673-2
25753603	Sedentary behavior in individuals with diabetic chronic kidney disease and maintenance hemodialysis	Anderton N, Giri A, Wei G, Marcus RL, Chen X, Bjordahl T, Habib A, Herrera J, Beddhu S	J Ren Nutr. 2015 Jul;25(4):364-70. doi: 10.1053/j.jrn.2015.01.018. Epub 2015 Mar 5	Anderton N	J Ren Nutr	2015	2015/3/11	PMC4469533	NIHMS657720	10.1053/j.jrn.2015.01.018
40576232	Dietary plant protein intake alleviates the adverse effect of sedentary behavior on chronic kidney disease incidence	Xia Z, Xiong Y	Nutr Hosp. 2025 Sep 4;42(4):772-779. doi: 10.20960/nh.05879	Xia Z	Nutr Hosp	2025	2025/6/27	-	-	10.20960/nh.05879
36415443	Chronic kidney disease: its relationship with obesity	Prasad R, Jha RK, Keerti A	Cureus. 2022 Oct 21;14(10):e30535. doi: 10.7759/cureus.30535. eCollection 2022 Oct	Prasad R	Cureus	2022	2022/11/23	PMC9675899		10.7759/cureus.30535
38727591	An intervention to reduce sedentary behavior in adults with chronic kidney disease: a feasibility study	Hannan MF, Sawatpanich A, Kringle E, Rivera E, Doorenbos AZ, Lash JP	Nephrol Nurs J. 2024 Mar-Apr;51(2):153-163	Hannan MF	Nephrol Nurs J	2024	2024/5/10	-	-	-
40085159	Prescribing physical activity and exercise for people with CKD: a practical guide by the global renal exercise network	Wilkinson TJ, Tarca B, Lightfoot CJ, Viana JL, Wilund KR, Ribeiro HS, Greenwood S, Sakkas GK, Kistler BM	Clin J Am Soc Nephrol. 2025 Mar 14;20(6):876-888. doi: 10.2215/CJN.0000000708	Wilkinson TJ	Clin J Am Soc Nephrol	2025	2025/3/14	PMC12160954		10.2215/CJN.0000000708
34935333	The influence of sedentary behavior on the relationship between cognitive function and vascular function in older adults with and without chronic kidney disease	Hannan M, Collins EG, Phillips SA, Quinn L, Steffen A, Bronas UG	Nephrol Nurs J. 2021 Nov-Dec;48(6):553-561	Hannan M	Nephrol Nurs J	2021	2021/12/22	PMC9113049	NIHMS1796638	
34450233	Sedentary behavior and estimated nephron number in middle-aged and older adults with or without chronic kidney disease	Kosaki K, Takahashi K, Matsui M, Yoshioka M, Mori S, Nishitani N, Shibata A, Saito C, Kuro-O M, Yamagata K, Oka K, Maeda S	Exp Gerontol. 2021 Oct 15;154:111531. doi: 10.1016/j.exger.2021.111531. Epub 2021 Aug 24	Kosaki K	Exp Gerontol	2021	2021/8/27	-	-	10.1016/j.exger.2021.111531
33774676	Physical activity, sedentary behavior, and skeletal muscle strength in patients with chronic kidney disease: an isotemporal substitution approach	Yoshioka M, Kosaki K, Matsui M, Takahashi K, Shibata A, Oka K, Kuro-O M, Saito C, Yamagata K, Maeda S	Phys Ther. 2021 Jul 1;101(7):pzab101. doi: 10.1093/ptj/pzab101	Yoshioka M	Phys Ther	2021	2021/3/28	-	-	10.1093/ptj/pzab101
34044686	Sedentary behavior in older adults with preclinical cognitive impairment with and without chronic kidney disease	Hannan M, Collins EG, Phillips SA, Quinn L, Steffen AD, Bronas UG	J Gerontol Nurs. 2021 Jun;47(6):35-42. doi: 10.3928/00989134-20210510-02. Epub 2021 Jun 1	Hannan M	J Gerontol Nurs	2021	2021/5/28	PMC8670529	NIHMS1758044	10.3928/00989134-20210510-02
39608749	The Sit Less, Interact and Move More (SLIMM-2) trial: Protocol for a randomized control trial of a sedentary behavior intervention, resistance training and semaglutide on sedentary behavior in persons with chronic kidney disease	Christensen JC, Anand S, Chertow GM, Lyden K, Sarwal A, Bjordahl T, Boucher R, Mohammed A, Oro EG, Akramimoghaddam F, Katkam N, Takyi A, Bissada G, Chakravartula AR, Lee E, Zheng A, Wei G, Greene T, Beddhu S	Contemp Clin Trials. 2025 Feb;149:107766. doi: 10.1016/j.cct.2024.107766. Epub 2024 Nov 26	Christensen JC	Contemp Clin Trials	2025	2024/11/28	PMC11875690	NIHMS2059898	10.1016/j.cct.2024.107766
36983870	Malnutrition patterns in children with chronic kidney disease	Karava V, Dotis J, Kondou A, Printza N	Life (Basel). 2023 Mar 6;13(3):713. doi: 10.3390/life13030713	Karava V	Life (Basel)	2023	2023/3/29	PMC10053690		10.3390/life13030713
32542048	Associations of accelerometer-measured physical activity and sedentary time with chronic kidney disease: the Framingham Heart Study	Lee J, Walker ME, Gabriel KP, Vasan RS, Xanthakis V	PLoS One. 2020 Jun 15;15(6):e0234825. doi: 10.1371/journal.pone.0234825. eCollection 2020	Lee J	PLoS One	2020	2020/6/17	PMC7295223		10.1371/journal.pone.0234825
38952392	Associations of accelerometer-measured physical activity and sedentary time with renal function and chronic kidney disease: a national population-based study	Suo X, Liu Y, Amoah AN, Bo Y, Lyu Q	Front Endocrinol (Lausanne). 2024 Jun 17;15:1403998. doi: 10.3389/fendo.2024.1403998. eCollection 2024	Suo X	Front Endocrinol (Lausanne)	2024	2024/7/2	PMC11215116		10.3389/fendo.2024.1403998
29989467	Impact of sedentary time on chronic kidney disease and disability incidence in community-dwelling Japanese older adults: a 4-year prospective cohort study	Lee S, Lee S, Bae S, Harada K, Jung S, Makino K, Shimada H	J Aging Phys Act. 2019 Apr 1;27(2):184-190. doi: 10.1123/japa.2017-0326. Epub 2019 Jan 16	Lee S	J Aging Phys Act	2019	2018/7/11	-	-	10.1123/japa.2017-0326
41101099	Association between chronic kidney disease and sarcopenia: a national cohort study and two-step Mendelian randomization analysis	Wang H, Zhou Y, Chen C, Zhang L, Zhang G	Arch Gerontol Geriatr. 2026 Jan;140:106051. doi: 10.1016/j.archger.2025.106051. Epub 2025 Oct 8	Wang H	Arch Gerontol Geriatr	2026	2025/10/16	-	-	10.1016/j.archger.2025.106051
32450794	Chronic kidney disease in Russia: the Ural eye and Medical Study	Bikbov MM, Zainullin RM, Kazakbaeva GM, Gilmanshin TR, Rakhimova EM, Rusakova IA, Bolshakova NI, Safiullina KR, Panda-Jonas S, Yakupova DF, Nikitin NA, Jonas JB	BMC Nephrol. 2020 May 25;21(1):198. doi: 10.1186/s12882-020-01843-4	Bikbov MM	BMC Nephrol	2020	2020/5/27	PMC7249426		10.1186/s12882-020-01843-4
39609014	Mediating role of inflammatory biomarkers on the association of physical activity, sedentary behaviour with chronic kidney disease: a cross-sectional study in NHANES 2007-2018	Peng P, Liu Z	BMJ Open. 2024 Nov 27;14(11):e084920. doi: 10.1136/bmjopen-2024-084920	Peng P	BMJ Open	2024	2024/11/28	PMC11603691		10.1136/bmjopen-2024-084920
33777360	Assessing physical activity and function in patients with chronic kidney disease: a narrative review	Bakker EA, Zoccali C, Dekker FW, Eijsvogels TMH, Jager KJ	Clin Kidney J. 2020 Sep 8;14(3):768-779. doi: 10.1093/ckj/sfaa156. eCollection 2021 Mar	Bakker EA	Clin Kidney J	2020	2021/3/29	PMC7986327		10.1093/ckj/sfaa156
37055762	Correction to: association between sedentary behavior and depression in US adults with chronic kidney disease: NHANES 2007-2018	Liu L, Yan Y, Qiu J, Chen Q, Zhang Y, Liu Y, Zhong X, Liu Y, Tan R	BMC Psychiatry. 2023 Apr 13;23(1):249. doi: 10.1186/s12888-023-04707-x	Liu L	BMC Psychiatry	2023	2023/4/13	PMC10103385		10.1186/s12888-023-04707-x
34532711	Sedentary behavior and change in kidney function: the Hispanic Community Health Study/Study of Latinos (HCHS/SOL)	Hannan M, Ricardo AC, Cai J, Franceschini N, Kaplan R, Marquez DX, Rosas SE, Schneiderman N, Sotres-Alvarez D, Talavera GA, Daviglus ML, Lash JP	Kidney360. 2021 Feb;2(2):245-253. doi: 10.34067/KID.0006202020. Epub 2021 Feb 25	Hannan M	Kidney360	2021	2021/9/17	PMC8443247	NIHMS1736559	10.34067/KID.0006202020
40758258	Association between sedentary behavior, hyperuricemia, and gout in American adults: a nationally representative cross-sectional study	Chen D, Li Y, Zhuang Y, Wang Y, Ye Y, Xue E, Chen Y, Zhao J	Clin Rheumatol. 2025 Oct;44(10):4249-4261. doi: 10.1007/s10067-025-07620-8. Epub 2025 Aug 4	Chen D	Clin Rheumatol	2025	2025/8/4	-	-	10.1007/s10067-025-07620-8
39181192	Association between physical activity & sedentary time on frailty in adults with chronic kidney disease: cross-sectional NHANES study	Zeng G, Lin Y, Xie P, Lin J, He Y, Wei J	Exp Gerontol. 2024 Oct 1;195:112557. doi: 10.1016/j.exger.2024.112557. Epub 2024 Aug 24	Zeng G	Exp Gerontol	2024	2024/8/24	-	-	10.1016/j.exger.2024.112557
35252275	Daily step counts in patients with chronic kidney disease: a systematic review and meta-analysis of observational studies	Zhang F, Ren Y, Wang H, Bai Y, Huang L	Front Med (Lausanne). 2022 Feb 17;9:842423. doi: 10.3389/fmed.2022.842423. eCollection 2022	Zhang F	Front Med (Lausanne)	2022	2022/3/7	PMC8891233		10.3389/fmed.2022.842423
34657911	Moderate-to-vigorous physical activity and sedentary behavior are independently associated with renal function: a cross-sectional study	Hara M, Nishida Y, Tanaka K, Shimanoe C, Koga K, Furukawa T, Higaki Y, Shinchi K, Ikezaki H, Murata M, Takeuchi K, Tamura T, Hishida A, Tsukamoto M, Kadomatsu Y, Matsuo K, Oze I, Mikami H, Kusakabe M, Takezaki T, Ibusuki R, Suzuki S, Nakagawa-Senda H, Matsui D, Koyama T, Kuriki K, Takashima N, Nakamura Y, Arisawa K, Katsuura-Kamano S, Wakai K	J Epidemiol. 2023 Jun 5;33(6):285-293. doi: 10.2188/jea.JE20210155. Epub 2022 Feb 22	Hara M	J Epidemiol	2023	2021/10/18	PMC10165219		10.2188/jea.JE20210155
33888536	Targeting sedentary behavior in CKD: a pilot and feasibility randomized controlled trial	Lyden K, Boucher R, Wei G, Zhou N, Christensen J, Chertow GM, Greene T, Beddhu S	Clin J Am Soc Nephrol. 2021 May 8;16(5):717-726. doi: 10.2215/CJN.12300720. Epub 2021 Apr 22	Lyden K	Clin J Am Soc Nephrol	2021	2021/4/23	PMC8259480		10.2215/CJN.12300720
26056174	Motivations and barriers to exercise in chronic kidney disease: a qualitative study	Clarke AL, Young HM, Hull KL, Hudson N, Burton JO, Smith AC	Nephrol Dial Transplant. 2015 Nov;30(11):1885-92. doi: 10.1093/ndt/gfv208. Epub 2015 Jun 7	Clarke AL	Nephrol Dial Transplant	2015	2015/6/10	-	-	10.1093/ndt/gfv208
41146863	Association of physical activity and sedentary behavior with stages of cardiovascular-kidney-metabolic syndrome among U.S. adults: NHANES 2007-2020	Pang S, Dongye Y, Bi Y, Wang J	Am Heart J Plus. 2025 Oct 14;60:100639. doi: 10.1016/j.ahjo.2025.100639. eCollection 2025 Dec	Pang S	Am Heart J Plus	2025	2025/10/28	PMC12554204		10.1016/j.ahjo.2025.100639
27295943	Association between sedentary time and kidney function in community-dwelling elderly Japanese people	Lee S, Shimada H, Lee S, Makizako H, Doi T, Harada K, Bae S, Harada K, Hotta R, Tsutsumimoto K, Yoshida D, Nakakubo S, Anan Y, Park H, Suzuki T	Geriatr Gerontol Int. 2017 May;17(5):730-736. doi: 10.1111/ggi.12779. Epub 2016 Jun 14	Lee S	Geriatr Gerontol Int	2017	2016/6/15	-	-	10.1111/ggi.12779
33888537	Moving beyond sedentarism in CKD	Kim TY, Roshanravan B	Clin J Am Soc Nephrol. 2021 May 8;16(5):674-676. doi: 10.2215/CJN.03460321. Epub 2021 Apr 22	Kim TY	Clin J Am Soc Nephrol	2021	2021/4/23	PMC8259495		10.2215/CJN.03460321
24950475	Impact of chronic kidney disease on quality of life, lung function, and functional capacity	Teixeira CG, Duarte Mdo C, Prado CM, Albuquerque EC, Andrade LB	J Pediatr (Rio J). 2014 Nov-Dec;90(6):580-6. doi: 10.1016/j.jped.2014.03.002. Epub 2014 Jun 17	Teixeira CG	J Pediatr (Rio J)	2014	2014/6/21	-	-	10.1016/j.jped.2014.03.002
35130637	Sedentarism, a modifiable risk factor for developing chronic kidney disease in healthy people	Concha AT, Mendoza FAR	Korean J Fam Med. 2022 Jan;43(1):27-36. doi: 10.4082/kjfm.20.0172. Epub 2022 Jan 20	Concha AT	Korean J Fam Med	2022	2022/2/7	PMC8820967		10.4082/kjfm.20.0172
31725147	Prevalence and correlates of physical activity across kidney disease stages: an observational multicentre study	Wilkinson TJ, Clarke AL, Nixon DGD, Hull KL, Song Y, Burton JO, Yates T, Smith AC	Nephrol Dial Transplant. 2021 Mar 29;36(4):641-649. doi: 10.1093/ndt/gfz235	Wilkinson TJ	Nephrol Dial Transplant	2021	2019/11/15	-	-	10.1093/ndt/gfz235
36567072	High physical activity alleviates the adverse effect of higher sedentary time on the incidence of chronic kidney disease	Oh W, Cho M, Jung SW, Moon JY, Lee SH, Hwang YC, Kim YG	J Cachexia Sarcopenia Muscle. 2023 Feb;14(1):622-631. doi: 10.1002/jcsm.13167. Epub 2022 Dec 25	Oh W	J Cachexia Sarcopenia Muscle	2023	2022/12/25	PMC9891972		10.1002/jcsm.13167
40120766	Time reallocation to moderate-to-vigorous physical activity and its association with chronic kidney disease prevalence in Chinese adults with type 2 diabetes	Xu M, Xu T, Li J, Zhang P, Wang H, Wang Y, Li L	Diabetes Res Clin Pract. 2025 Apr;222:112116. doi: 10.1016/j.diabres.2025.112116. Epub 2025 Mar 20	Xu M	Diabetes Res Clin Pract	2025	2025/3/22	-	-	10.1016/j.diabres.2025.112116
37837705	Association of physical activity with endothelial dysfunction among adults with and without chronic kidney disease: the Maastricht study	Bellos I, Marinaki S, Lagiou P, Boletis IN, Stehouwer CDA, van Greevenbroek MMJ, Eussen SJPM, de Galan BE, Savelberg HHCM, Koster A, Wesselius A, Benetou V	Atherosclerosis. 2023 Oct;383:117330. doi: 10.1016/j.atherosclerosis.2023.117330. Epub 2023 Oct 5	Bellos I	Atherosclerosis	2023	2023/10/14	-	-	10.1016/j.atherosclerosis.2023.117330
26077152	Risk factors and their interaction on chronic kidney disease: a multi-centre case control study in Taiwan	Su SL, Lin C, Kao S, Wu CC, Lu KC, Lai CH, Yang HY, Chiu YL, Chen JS, Sung FC, Ko YC, Lee CT, Yang Y, Yang CW, Hwang SJ, Wang MC, Hsu YH, Wu MY, Hsueh YM, Chiou HY, Lin YF	BMC Nephrol. 2015 Jun 16;16:83. doi: 10.1186/s12882-015-0065-x	Su SL	BMC Nephrol	2015	2015/6/17	PMC4469431		10.1186/s12882-015-0065-x
32852701	Occupational sedentary behavior and prediction of proteinuria in young to middle-aged adults: a retrospective cohort study	Fujii Y, Yamamoto R, Shinzawa M, Kimura Y, Aoki K, Tomi R, Ozaki S, Yoshimura R, Taneike M, Nakanishi K, Nishida M, Yamauchi-Takihara K, Kudo T, Isaka Y, Moriyama T	J Nephrol. 2021 Jun;34(3):719-728. doi: 10.1007/s40620-020-00826-w. Epub 2020 Aug 27	Fujii Y	J Nephrol	2021	2020/8/28	-	-	10.1007/s40620-020-00826-w
20740391	Television viewing time and risk of chronic kidney disease in adults: the AusDiab study	Lynch BM, White SL, Owen N, Healy GN, Chadban SJ, Atkins RC, Dunstan DW	Ann Behav Med. 2010 Dec;40(3):265-74. doi: 10.1007/s12160-010-9209-1	Lynch BM	Ann Behav Med	2010	2010/8/27	-	-	10.1007/s12160-010-9209-1
19820134	Physical activity and mortality in chronic kidney disease (NHANES III)	Beddhu S, Baird BC, Zitterkoph J, Neilson J, Greene T	Clin J Am Soc Nephrol. 2009 Dec;4(12):1901-6. doi: 10.2215/CJN.01970309. Epub 2009 Oct 9	Beddhu S	Clin J Am Soc Nephrol	2009	2009/10/13	PMC2798872		10.2215/CJN.01970309
34026868	Metabolism and chronic inflammation: the links between chronic heart failure and comorbidities	Li Z, Zhao H, Wang J	Front Cardiovasc Med. 2021 May 5;8:650278. doi: 10.3389/fcvm.2021.650278. eCollection 2021	Li Z	Front Cardiovasc Med	2021	2021/5/24	PMC8131678		10.3389/fcvm.2021.650278
34319455	Replacing sedentary time for physical activity on bone density in patients with chronic kidney disease	Yoshioka M, Kosaki K, Matsui M, Shibata A, Oka K, Kuro-O M, Saito C, Yamagata K, Maeda S	J Bone Miner Metab. 2021 Nov;39(6):1091-1100. doi: 10.1007/s00774-021-01255-w. Epub 2021 Jul 28	Yoshioka M	J Bone Miner Metab	2021	2021/7/28	-	-	10.1007/s00774-021-01255-w
26684326	Physical activity and screen time in adolescents in the chronic kidney disease in children (CKiD) cohort	Clark SL, Denburg MR, Furth SL	Pediatr Nephrol. 2016 May;31(5):801-8. doi: 10.1007/s00467-015-3287-z. Epub 2015 Dec 18	Clark SL	Pediatr Nephrol	2016	2015/12/20	PMC4924924	NIHMS796487	10.1007/s00467-015-3287-z
36126645	Long-term trends in risk factor management in respondents with chronic kidney disease in the USA	Liu Q, Bai B, Liu F, Chen Y, Wang Y, Wang H, Ma H, Geng Q	Am J Nephrol. 2022;53(8-9):614-623. doi: 10.1159/000525733. Epub 2022 Sep 20	Liu Q	Am J Nephrol	2022	2022/9/20	-	-	10.1159/000525733
41223326	Self-reported vs. device-measured sedentary time in adults with chronic kidney disease	Hannan MF, Sawatpanich A, Kringle E, Rivera E, Doorenbos AZ, Lash JP	Nephrol Nurs J. 2025 Sep-Oct;52(5):509-514	Hannan MF	Nephrol Nurs J	2025	2025/11/12	-	-	-
25346108	Physical activity and risk of end-stage kidney disease in the Singapore Chinese health study	Jafar TH, Jin A, Koh WP, Yuan JM, Chow KY	Nephrology (Carlton). 2015 Feb;20(2):61-7. doi: 10.1111/nep.12355	Jafar TH	Nephrology (Carlton)	2015	2014/10/28	PMC4495910	NIHMS638341	10.1111/nep.12355
38172696	Objectively measured daily steps as an outcome in a clinical trial of chronic kidney disease: a systematic review	Huang L, Wang H, Bai Y, Zhang H, Zhang F, Zhong Y	BMC Nephrol. 2024 Jan 3;25(1):10. doi: 10.1186/s12882-023-03412-x	Huang L	BMC Nephrol	2024	2024/1/3	PMC10765814		10.1186/s12882-023-03412-x
36837547	Oral anticoagulation in patients with chronic liver disease	Costache RS, Dragomirică AS, Gheorghe BE, Balaban VD, Stanciu SM, Jinga M, Costache DO	Medicina (Kaunas). 2023 Feb 12;59(2):346. doi: 10.3390/medicina59020346	Costache RS	Medicina (Kaunas)	2023	2023/2/25	PMC9967228		10.3390/medicina59020346
40672525	Exercise therapy to improve mobility, active behaviour and quality of life of chronic kidney disease patients with peripheral artery disease: study protocol for the EXACT-CKDPAD multicentre randomised controlled trial	Manfredini F, Panuccio V, Battaglia Y, Storari A, Lamberti N, Piva G, Veronesi M, Tripepi R, Rinaldo N, Crepaldi A, Momentè C, Piccinini A, Traina L, Fargion AT, Straudi S, Baroni A, De Giorgi A, Martinuzzi C, Monesi M, Capitanini A, Aucella F, Cupisti A, Mallamaci F, Zoccali C, Manfredini R	BMJ Open Sport Exerc Med. 2025 Jul 15;11(3):e002740. doi: 10.1136/bmjsem-2025-002740. eCollection 2025	Manfredini F	BMJ Open Sport Exerc Med	2025	2025/7/17	PMC12265826		10.1136/bmjsem-2025-002740
37885172	The impact of sedentary behavior on renal function decline in 132,123 middle aged and older adults: a nationwide cohort study	Yu P, Zhao Z, Huang L, Zou H, Meng X, Kan R, Yu X	Med Sci Monit. 2023 Oct 27;29:e941111. doi: 10.12659/MSM.941111	Yu P	Med Sci Monit	2023	2023/10/27	PMC10617246		10.12659/MSM.941111
29968886	Determinants of quality of life as measured with variants of SF-36 in patients with predialysis chronic kidney disease	Alhaji MM, Tan J, Hamid MR, Timbuak JA, Naing L, Tuah NA	Saudi Med J. 2018 Jul;39(7):653-661. doi: 10.15537/smj.2018.7.21352	Alhaji MM	Saudi Med J	2018	2018/7/4	PMC6146254		10.15537/smj.2018.7.21352
38498271	Phosphate is associated with frailty in older patients with chronic kidney disease not on dialysis	Veloso MP, Coelho VA, Sekercioglu N, Moyses RMA, Elias RM	Int Urol Nephrol. 2024 Aug;56(8):2725-2731. doi: 10.1007/s11255-024-03985-y. Epub 2024 Mar 18	Veloso MP	Int Urol Nephrol	2024	2024/3/18	-	-	10.1007/s11255-024-03985-y
40785651	Physical activity, sedentary behavior, and cardiometabolic risk factors in people living with HIV	Jones R, Hankes MJ, Armstrong ND, Pettee Gabriel K, Buford TW, Dooley EE	Behav Med. 2025 Aug 11:1-10. doi: 10.1080/08964289.2025.2543263. Online ahead of print	Jones R	Behav Med	2025	2025/8/11	PMC12452992	NIHMS2107464	10.1080/08964289.2025.2543263
26284458	Autonomic modulation analysis in active and sedentary kidney transplanted recipients	Moraes Dias CJ, Anaisse Azoubel LM, Araújo Costa H, Costa Maia E, Rodrigues B, Silva-Filho AC, Dias-Filho CA, Irigoyen MC, Leite RD, de Oliveira Junior MS, Mostarda CT	Clin Exp Pharmacol Physiol. 2015 Dec;42(12):1239-44. doi: 10.1111/1440-1681.12481	Moraes Dias CJ	Clin Exp Pharmacol Physiol	2015	2015/8/19	-	-	10.1111/1440-1681.12481
31141443	Physical activity intensity and biopsychosocial correlates in Chinese middle-aged and older adults with chronic kidney disease	Chen J, Nicklett EJ, He Y, Lou VW	J Aging Phys Act. 2019 Dec 1;27(4):871-878. doi: 10.1123/japa.2018-0078	Chen J	J Aging Phys Act	2019	2019/5/30	-	-	10.1123/japa.2018-0078
40834545	Healthy lifestyles and risk of major non-communicable chronic diseases and mortality in individuals with metabolic syndrome	Gao X, Zheng J, Liu M, Liu C, Wang Y, Li Y, Chen W, Li X, Wang H	J Nutr Health Aging. 2025 Sep;29(9):100654. doi: 10.1016/j.jnha.2025.100654. Epub 2025 Aug 19	Gao X	J Nutr Health Aging	2025	2025/8/20	PMC12396011		10.1016/j.jnha.2025.100654
25731886	Mediators of the association between low socioeconomic status and chronic kidney disease in the United States	Vart P, Gansevoort RT, Crews DC, Reijneveld SA, Bültmann U	Am J Epidemiol. 2015 Mar 15;181(6):385-96. doi: 10.1093/aje/kwu316. Epub 2015 Mar 1	Vart P	Am J Epidemiol	2015	2015/3/4	PMC4425833		10.1093/aje/kwu316
26786187	Blood pressure response to acute and chronic exercise in chronic kidney disease	Headley S, Germain M, Wood R, Joubert J, Milch C, Evans E, Cornelius A, Brewer B, Taylor B, Pescatello LS	Nephrology (Carlton). 2017 Jan;22(1):72-78. doi: 10.1111/nep.12730	Headley S	Nephrology (Carlton)	2017	2016/1/21	-	-	10.1111/nep.12730
40796264	Type 2 diabetes in South Asians	Misra A, Sattar N, Ghosh A, Nassar M, Jayawardena R, Gupta R	BMJ. 2025 Aug 12;390:e079801. doi: 10.1136/bmj-2024-079801	Misra A	BMJ	2025	2025/8/12	-	-	10.1136/bmj-2024-079801
29617428	Amount and pattern of physical activity and sedentary behavior are associated with kidney function and kidney damage: the Maastricht study	Martens RJH, van der Berg JD, Stehouwer CDA, Henry RMA, Bosma H, Dagnelie PC, van Dongen MCJM, Eussen SJPM, Schram MT, Sep SJS, van der Kallen CJH, Schaper NC, Savelberg HHCM, van der Sande FM, Kroon AA, Kooman JP, Koster A	PLoS One. 2018 Apr 4;13(4):e0195306. doi: 10.1371/journal.pone.0195306. eCollection 2018	Martens RJH	PLoS One	2018	2018/4/5	PMC5884554		10.1371/journal.pone.0195306
34684396	Energy requirement for elderly CKD patients	D'Alessandro C, Giannese D, Avino M, Cupisti A	Nutrients. 2021 Sep 27;13(10):3396. doi: 10.3390/nu13103396	D'Alessandro C	Nutrients	2021	2021/10/23	PMC8541480		10.3390/nu13103396
33826736	Causal effects of physical activity or sedentary behaviors on kidney function: an integrated population-scale observational analysis and Mendelian randomization study	Park S, Lee S, Kim Y, Lee Y, Kang MW, Kim K, Kim YC, Han SS, Lee H, Lee JP, Wook Joo K, Lim CS, Kim YS, Kim DK.	Nephrol Dial Transplant. 2022 May 25;37(6):1059-1068. doi: 10.1093/ndt/gfab153	Park S	Nephrol Dial Transplant	2022	2021/4/7	-	-	10.1093/ndt/gfab153
29482935	Physical activity in kidney transplant recipients: a review	Takahashi A, Hu SL, Bostom A	Am J Kidney Dis. 2018 Sep;72(3):433-443. doi: 10.1053/j.ajkd.2017.12.005. Epub 2018 Feb 23	Takahashi A	Am J Kidney Dis	2018	2018/2/28	-	-	10.1053/j.ajkd.2017.12.005
41040866	Global, regional, and national burden of type 2 diabetes-related diabetic kidney disease attributable to low physical activity from 1990 to 2021: a systematic analysis of the Global Burden of Disease Study 2021 with predictions to 2050	Wang M, Yan R, Xia W, Gao Y, Liu Y, Bao L, Luo H, E J, Wang H, Li B, Zheng Y	Front Endocrinol (Lausanne). 2025 Sep 17;16:1625973. doi: 10.3389/fendo.2025.1625973. eCollection 2025	Wang M	Front Endocrinol (Lausanne)	2025	2025/10/3	PMC12483903		10.3389/fendo.2025.1625973
40296017	Global trends in chronic kidney disease mortality and disability-adjusted life years attributable to low physical activity (1990-2021): a growing public health challenge	Zhao Z, Mi J, Jin H, Li S, Bai X	BMC Nephrol. 2025 Apr 28;26(1):215. doi: 10.1186/s12882-025-04117-z	Zhao Z	BMC Nephrol	2025	2025/4/28	PMC12039278		10.1186/s12882-025-04117-z
22258539	Physical activity in the prevention of chronic kidney disease	Stump CS	Cardiorenal Med. 2011;1(3):164-173. doi: 10.1159/000329929. Epub 2011 Jul 25	Stump CS	Cardiorenal Med	2011	2012/1/20	PMC3150958		10.1159/000329929
34338337	Care and prevention during the COVID-19 pandemic quarantine: sedentary lifestyle and increased risk of kidney stones	Aleebrahim-Dehkordi E, Soleiman-Dehkordi E, Saberianpour S, Hasanpour-Dehkordi A, Hasanpour Dehkordi A	Przegl Epidemiol. 2021;75(1):45-50. doi: 10.32394/pe.75.04	Aleebrahim-Dehkordi E	Przegl Epidemiol	2021	2021/8/2	-	-	10.32394/pe.75.04
33959264	The PrEscription of intraDialytic exercise to improve quAlity of Life in patients with chronic kidney disease trial: study design and baseline data for a multicentre randomized controlled trial	Greenwood SA, Koufaki P, Macdonald J, Bhandari S, Burton J, Dasgupta I, Farrington K, Ford I, Kalra PA, Kean S, Kumwenda M, Macdougall IC, Messow CM, Mitra S, Reid C, Smith AC, Taal MW, Thomson PC, Wheeler DC, White C, Yaqoob M, Mercer TH	Clin Kidney J. 2020 Sep 10;14(5):1345-1355. doi: 10.1093/ckj/sfaa107. eCollection 2021 May	Greenwood SA	Clin Kidney J	2020	2021/5/7	PMC8087141		10.1093/ckj/sfaa107
26453919	High intensity interval training favourably affects antioxidant and inflammation mRNA expression in early-stage chronic kidney disease	Tucker PS, Briskey DR, Scanlan AT, Coombes JS, Dalbo VJ	Free Radic Biol Med. 2015 Dec;89:466-72. doi: 10.1016/j.freeradbiomed.2015.07.162. Epub 2015 Oct 8	Tucker PS	Free Radic Biol Med	2015	2015/10/11	-	-	10.1016/j.freeradbiomed.2015.07.162
31471443	The association of exercise and sedentary behaviours with incident end-stage renal disease: the Southern Community Cohort Study	Pike M, Taylor J, Kabagambe E, Stewart TG, Robinson-Cohen C, Morse J, Akwo E, Abdel-Kader K, Siew ED, Blot WJ, Ikizler TA, Lipworth L	BMJ Open. 2019 Aug 30;9(8):e030661. doi: 10.1136/bmjopen-2019-030661	Pike M	BMJ Open	2019	2019/9/1	PMC6720137		10.1136/bmjopen-2019-030661
39257785	Six months of physical inactivity is insufficient to cause chronic kidney disease in C57BL/6J mice	Opurum PC, Decker ST, Stuart D, Peterlin AD, Paula VL, Siripoksup P, Drummond MJ, Sanchez A, Ramkumar N, Funai K	bioRxiv [Preprint]. 2024 Aug 30:2024.08.29.610415. doi: 10.1101/2024.08.29.610415	Opurum PC	bioRxiv	2024	2024/9/11	PMC11384017		10.1101/2024.08.29.610415
26282583	Objectively measured sedentary time, physical activity and kidney function in people with recently diagnosed type 2 diabetes: a prospective cohort analysis	Guo VY, Brage S, Ekelund U, Griffin SJ, Simmons RK; ADDITION-Plus study team	Diabet Med. 2016 Sep;33(9):1222-9. doi: 10.1111/dme.12886. Epub 2015 Sep 8	Guo VY	Diabet Med	2016	2015/8/19	PMC5017300		10.1111/dme.12886
38300434	Diagnostic accuracy of step count as an indicator of sedentary behavior in patients with end-stage kidney disease on hemodialysis	Jesus LADS, Pinheiro BV, Alvarenga BA, Paticcié GF, Oliveira CC, Lucinda LMF, Reboredo MM	J Nephrol. 2024 Apr;37(3):777-779. doi: 10.1007/s40620-023-01881-9. Epub 2024 Feb 1	Jesus LADS	J Nephrol	2024	2024/2/1	-	-	10.1007/s40620-023-01881-9
24643902	Diabetes and associated complications in the South Asian population	Shah A, Kanaya AM	Curr Cardiol Rep. 2014 May;16(5):476. doi: 10.1007/s11886-014-0476-5	Shah A	Curr Cardiol Rep	2014	2014/3/20	PMC4026332	NIHMS577365	10.1007/s11886-014-0476-5
41048221	Global burden of chronic kidney disease due to diabetes mellitus type 2 attributable to low physical activity and high body mass index from 1990 to 2021	Pan W, Li F, Du X, Ye M, Wang S, Shi K	Biol Sport. 2025 May 8;42(4):171-186. doi: 10.5114/biolsport.2025.150045. eCollection 2025 Oct	Pan W	Biol Sport	2025	2025/10/6	PMC12492348		10.5114/biolsport.2025.150045
25117619	The role of physical activity in the CKD setting	Aucella F, Valente GL, Catizone L	Kidney Blood Press Res. 2014;39(2-3):97-106. doi: 10.1159/000355783. Epub 2014 Jul 29	Aucella F	Kidney Blood Press Res	2014	2014/8/14	-	-	10.1159/000355783
29265674	Aerobic exercise training rescues protein quality control disruption on white skeletal muscle induced by chronic kidney disease in rats	De Moraes WMAM, de Souza PRM, da Paixão NA, de Sousa LGO, Ribeiro DA, Marshall AG, Prestes J, Irigoyen MC, Brum PC, Medeiros A	J Cell Mol Med. 2018 Mar;22(3):1452-1463. doi: 10.1111/jcmm.13374. Epub 2017 Dec 19	De Moraes WMAM	J Cell Mol Med	2018	2017/12/22	PMC5824409		10.1111/jcmm.13374
29162432	Renal damage in the metabolic syndrome (MetSx): disorders implicated	Joyce T, Chirino YI, Natalia MT, Jose PC	Eur J Pharmacol. 2018 Jan 5;818:554-568. doi: 10.1016/j.ejphar.2017.11.032. Epub 2017 Nov 21	Joyce T	Eur J Pharmacol	2018	2017/11/23	-	-	10.1016/j.ejphar.2017.11.032
40327334	Combined sedentarism and high-fat diet induce early signs of kidney injury in C57BL/6J mice	Opurum PC, Decker ST, Stuart D, Peterlin AD, Paula VL, Siripoksup P, Drummond MJ, Sanchez A, Ramkumar N, Funai K	Am J Physiol Renal Physiol. 2025 Jun 1;328(6):F850-F860. doi: 10.1152/ajprenal.00259.2024. Epub 2025 May 6	Opurum PC	Am J Physiol Renal Physiol	2025	2025/5/6	PMC12147876	NIHMS2083129	10.1152/ajprenal.00259.2024
30817720	Sedentary time and white matter hyperintensity volume in older adults	Bronas UG, Steffen A, Dion C, Boots EA, Arfanakis K, Marquez DX, Lamar M	Med Sci Sports Exerc. 2019 Aug;51(8):1613-1618. doi: 10.1249/MSS.0000000000001957	Bronas UG	Med Sci Sports Exerc	2019	2019/3/1	PMC7282192	NIHMS1594129	10.1249/MSS.0000000000001957
34575704	Physical inactivity: a modifiable risk factor for morbidity and mortality in kidney transplantation	Ponticelli C, Favi E	J Pers Med. 2021 Sep 18;11(9):927. doi: 10.3390/jpm11090927	Ponticelli C	J Pers Med	2021	2021/9/28	PMC8470604		10.3390/jpm11090927
28138130	Physical inactivity: a risk factor and target for intervention in renal care	Zelle DM, Klaassen G, van Adrichem E, Bakker SJ, Corpeleijn E, Navis G	Nat Rev Nephrol. 2017 Mar;13(3):152-168. doi: 10.1038/nrneph.2016.187. Epub 2017 Jan 31	Zelle DM	Nat Rev Nephrol	2017	2017/2/1	-	-	10.1038/nrneph.2016.187
39197432	Exercise, dialysis, and environment: a narrative review in an ecological perspective	Piva G, Storari A, Battaglia Y, Manfredini F, Lamberti N	Kidney Blood Press Res. 2024;49(1):773-786. doi: 10.1159/000540910. Epub 2024 Aug 28	Piva G	Kidney Blood Press Res	2024	2024/8/28	-	-	10.1159/000540910
30629045	Physical activity and cardiovascular risk among kidney transplant patients	Kang AW, Garber CE, Eaton CB, Risica PM, Bostom AG	Med Sci Sports Exerc. 2019 Jun;51(6):1154-1161. doi: 10.1249/MSS.0000000000001886	Kang AW	Med Sci Sports Exerc	2019	2019/1/11	PMC6522300	NIHMS1517969	10.1249/MSS.0000000000001886
32045878	Modifiable physical factors associated with physical functioning for patients receiving dialysis: a systematic review	Tarca BD, Wycherley TP, Bennett P, Meade A, Ferrar KE	J Phys Act Health. 2020 Apr 1;17(4):475-489. doi: 10.1123/jpah.2019-0338	Tarca BD	J Phys Act Health	2020	2020/2/12	-	-	10.1123/jpah.2019-0338
29909935	CKD and sedentary time: results from the Canadian health measures survey	Glavinovic T, Ferguson T, Komenda P, Rigatto C, Duhamel TA, Tangri N, Bohm C	Am J Kidney Dis. 2018 Oct;72(4):529-537. doi: 10.1053/j.ajkd.2018.03.031. Epub 2018 Jun 15	Glavinovic T	Am J Kidney Dis	2018	2018/6/19	-	-	10.1053/j.ajkd.2018.03.031
31595572	The brown bear as a translational model for sedentary lifestyle-related diseases	Fröbert O, Frøbert AM, Kindberg J, Arnemo JM, Overgaard MT	J Intern Med. 2020 Mar;287(3):263-270. doi: 10.1111/joim.12983. Epub 2019 Oct 24	Fröbert O	J Intern Med	2020	2019/10/10	-	-	10.1111/joim.12983
25503447	Impact of home-based aerobic exercise on the physical capacity of overweight patients with chronic kidney disease	Aoike DT, Baria F, Kamimura MA, Ammirati A, de Mello MT, Cuppari L	Int Urol Nephrol. 2015 Feb;47(2):359-67. doi: 10.1007/s11255-014-0894-8. Epub 2014 Dec 11	Aoike DT	Int Urol Nephrol	2015	2014/12/16	-	-	10.1007/s11255-014-0894-8
26090382	High intensity interval training favourably affects angiotensinogen mRNA expression and markers of cardiorenal health in a rat model of early-stage chronic kidney disease	Tucker PS, Scanlan AT, Dalbo VJ	Biomed Res Int. 2015;2015:156584. doi: 10.1155/2015/156584. Epub 2015 May 24	Tucker PS	Biomed Res Int	2015	2015/6/20	PMC4458272		10.1155/2015/156584
29427767	An assessment of the relationship of physical activity, obesity, and chronic diseases/conditions between active/obese and sedentary/ normal weight American women in a national sample	Pharr JR, Coughenour CA, Bungum TJ	Public Health. 2018 Mar;156:117-123. doi: 10.1016/j.puhe.2017.12.013. Epub 2018 Feb 20	Pharr JR	Public Health	2018	2018/2/11	-	-	10.1016/j.puhe.2017.12.013
24449105	Randomized controlled trial to evaluate the impact of aerobic exercise on visceral fat in overweight chronic kidney disease patients	Baria F, Kamimura MA, Aoike DT, Ammirati A, Rocha ML, de Mello MT, Cuppari L	Nephrol Dial Transplant. 2014 Apr;29(4):857-64. doi: 10.1093/ndt/gft529. Epub 2014 Jan 20	Baria F	Nephrol Dial Transplant	2014	2014/1/23	-	-	10.1093/ndt/gft529
27547714	Genomic integrity is favourably affected by high-intensity interval training in an animal model of early-stage chronic kidney disease	Tucker PS, Scanlan AT, Vella RK, Dalbo VJ	Sports Med Open. 2016 Aug 9;2:28. doi: 10.1186/s40798-016-0055-y. eCollection 2015 Jun-	Tucker PS	Sports Med Open	2016	2016/8/23	PMC4978751		10.1186/s40798-016-0055-y
41110464	The mediating effect of 24-hour movement behaviors on mortality risk in chronic kidney disease: a compositional mediation and life expectancy analysis	Wang Z, Huang Y, Xu J, Lawrence WR, Chen J, Cai Y, Zhang T, Mo Q, Gao Y, Lin Z	J Phys Act Health. 2025 Oct 17:1-8. doi: 10.1123/jpah.2025-0467. Online ahead of print	Wang Z	J Phys Act Health	2025	2025/10/19	-	-	10.1123/jpah.2025-0467
28513257	Factors associated with levels of physical activity in chronic kidney disease patients undergoing hemodialysis: the role of dialysis versus nondialysis day	da Costa Rosa CS, Nishimoto DY, Freitas Júnior IF, Ciolac EG, Monteiro HL	J Phys Act Health. 2017 Sep;14(9):726-732. doi: 10.1123/jpah.2016-0715. Epub 2017 May 17	da Costa Rosa CS	J Phys Act Health	2017	2017/5/18	-	-	10.1123/jpah.2016-0715
40976358	Obesity and inactivity cluster the strongest risk factor for the development of heart failure in a population-based study	van Essen BJ, Dan NAE, Tharsana GN, Kaur P, Emmens JE, Ouwerkerk W, Gansevoort RT, Bakker SJL, de Boer RA, Damman K, van Veldhuisen DJ, Voors AA, Tromp J	Int J Cardiol. 2026 Jan 1;442:133914. doi: 10.1016/j.ijcard.2025.133914. Epub 2025 Sep 19	van Essen BJ	Int J Cardiol	2026	2025/9/21	-	-	10.1016/j.ijcard.2025.133914
21200336	Association between physical activity and kidney function: National Health and Nutrition Examination Survey	Hawkins MS, Sevick MA, Richardson CR, Fried LF, Arena VC, Kriska AM	Med Sci Sports Exerc. 2011 Aug;43(8):1457-64. doi: 10.1249/MSS.0b013e31820c0130	Hawkins MS	Med Sci Sports Exerc	2011	2011/1/5	-	-	10.1249/MSS.0b013e31820c0130
23932846	Maladaptive immune and inflammatory pathways lead to cardiovascular insulin resistance	Aroor AR, McKarns S, Demarco VG, Jia G, Sowers JR	Metabolism. 2013 Nov;62(11):1543-52. doi: 10.1016/j.metabol.2013.07.001. Epub 2013 Aug 8	Aroor AR	Metabolism	2013	2013/8/13	PMC3809332	NIHMS504622	10.1016/j.metabol.2013.07.001
37122278	Cardiovascular risk factors in Sub-Saharan African women	Chastaingt L, Gbaguidi GN, Magne J, Preux PM, Aboyans V, Houinato D, Lacroix P; TAHES group	Vasa. 2023 May;52(3):186-192. doi: 10.1024/0301-1526/a001058. Epub 2023 Feb 22	Chastaingt L	Vasa	2023	2023/5/1	-	-	10.1024/0301-1526/a001058
33060457	Dietary protein interventions to improve nutritional status in end-stage renal disease patients undergoing hemodialysis	Hendriks FK, Kooman JP, van Loon LJC	Curr Opin Clin Nutr Metab Care. 2021 Jan;24(1):79-87. doi: 10.1097/MCO.0000000000000703	Hendriks FK	Curr Opin Clin Nutr Metab Care	2021	2020/10/16	PMC7752218		10.1097/MCO.0000000000000703
37286321	Assessing physical inactivity as a risk factor for chronic kidney diseases in Iranian population	Moeinzadeh F, Babahajiani M, Seirafian S, Mansourian M, Mortazavi M, Shahidi S, Vahdat S, Saleki M	BMJ Open. 2023 Jun 7;13(6):e070360. doi: 10.1136/bmjopen-2022-070360	Moeinzadeh F	BMJ Open	2023	2023/6/7	PMC10254705		10.1136/bmjopen-2022-070360
32493450	Sedentary behaviour, physical activity, and renal function in older adults: isotemporal substitution modelling	Kosaki K, Tanahashi K, Matsui M, Akazawa N, Osuka Y, Tanaka K, Dunstan DW, Owen N, Shibata A, Oka K, Maeda S	BMC Nephrol. 2020 Jun 3;21(1):211. doi: 10.1186/s12882-020-01869-8	Kosaki K	BMC Nephrol	2020	2020/6/5	PMC7268521		10.1186/s12882-020-01869-8
19801138	Anabolic and catabolic mechanisms in end-stage renal disease	Johansen KL	Adv Chronic Kidney Dis. 2009 Nov;16(6):501-10. doi: 10.1053/j.ackd.2009.07.007	Johansen KL	Adv Chronic Kidney Dis	2009	2009/10/6	-	-	10.1053/j.ackd.2009.07.007
37675341	Frailty as a dynamic process in a diverse cohort of older persons with dialysis-dependent CKD	Kutner NG, Zhang R.	Front Nephrol. 2023 Jan 20;3:1031338. doi: 10.3389/fneph.2023.1031338. eCollection 2023.	Kutner NG	Front Nephrol	2023	2023/9/7	PMC10479570		10.3389/fneph.2023.1031338
21488032	[Physical activity and renal transplantation]	Mosconi G, Roi GS, Nanni Costa A, Stefoni S	G Ital Nefrol. 2011 Mar-Apr;28(2):174-87	Mosconi G	G Ital Nefrol	2011	2011/4/14	-	-	-
28575141	Objectively measured physical activity and kidney function in older men; a cross-sectional population-based study	Parsons TJ, Sartini C, Ash S, Lennon LT, Wannamethee SG, Lee IM, Whincup PH, Jefferis BJ	Age Ageing. 2017 Nov 1;46(6):1010-1014. doi: 10.1093/ageing/afx091	Parsons TJ	Age Ageing	2017	2017/6/3	PMC5860422		10.1093/ageing/afx091
39462313	Independent and joint effects of self-reported physical activity and sedentary behaviors on mortality in community-dwelling older persons: a prospective cohort study	Lin CC, Li CI, Liu CS, Lin CH, Lin YC, Yang SY, Li TC	BMC Geriatr. 2024 Oct 26;24(1):886. doi: 10.1186/s12877-024-05493-1	Lin CC	BMC Geriatr	2024	2024/10/27	PMC11515286		10.1186/s12877-024-05493-1
37810519	An intervention to decrease sedentary behavior in older adults: A secondary analysis of a randomized controlled trial	Abraham N, Lyden K, Boucher R, Wei G, Gonce V, Carle J, Fornadi K, Supiano MA, Christensen J, Beddhu S	Obes Sci Pract. 2023 Jun 15;9(5):529-537. doi: 10.1002/osp4.687. eCollection 2023 Oct	Abraham N	Obes Sci Pract	2023	2023/10/9	PMC10551115		10.1002/osp4.687
27274994	Synergistic effects of six chronic disease pairs on decreased physical activity: the SMILE cohort study	Dörenkamp S, Mesters I, Vos R, Schepers J, van den Akker M, Teijink J, de Bie R	Biomed Res Int. 2016;2016:9427231. doi: 10.1155/2016/9427231. Epub 2016 May 5	Dörenkamp S	Biomed Res Int	2016	2016/6/9	PMC4871954		10.1155/2016/9427231
39433040	Nutrition and physical activity in older adults with CKD: two sides of the same coin	D'Alessandro C, Giannese D, Ruisi MR, Pellegrino N, Lucenteforte E, Panichi V, Cupisti A	Kidney Blood Press Res. 2024;49(1):978-986. doi: 10.1159/000541902. Epub 2024 Oct 21	D'Alessandro C	Kidney Blood Press Res	2024	2024/10/21	-	-	10.1159/000541902
34950045	Accelerometry correlates in body composition, physical fitness, and disease symptom burden: a pilot study in end-stage renal disease	Sember V, Bogataj Š, Ribeiro JC, Paravlić A, Pajek M, Pajek J	Front Physiol. 2021 Dec 7;12:737069. doi: 10.3389/fphys.2021.737069. eCollection 2021	Sember V	Front Physiol	2021	2021/12/24	PMC8688961		10.3389/fphys.2021.737069
22177601	[Is there a place for the physical activity in the prevention of the fractures of chronic kidney disease patients?]	Chauveau P, Lasseur C, Aparicio M	Nephrol Ther. 2012 Jul;8(4):216-9. doi: 10.1016/j.nephro.2011.10.004. Epub 2011 Dec 15	Chauveau P	Nephrol Ther	2012	2011/12/20	-	-	10.1016/j.nephro.2011.10.004
34979979	Association of accelerometer-measured physical activity with kidney function in a Japanese population: the DOSANCO Health Study	Sasaki S, Nakamura K, Ukawa S, Okada E, Amagasa S, Inoue S, Kimura T, Yoshimura A, Tanaka A, Nakagawa T, Imae A, Tamakoshi A	BMC Nephrol. 2022 Jan 3;23(1):7. doi: 10.1186/s12882-021-02635-0	Sasaki S	BMC Nephrol	2022	2022/1/4	PMC8722077		10.1186/s12882-021-02635-0
36512143	Associations of prolonged occupational sitting with the spectrum of kidney disease: results from a cohort of a half-million Asian adults	Tsai MK, Gao W, Chien KL, Baw CK, Hsu CC, Wen CP	Sports Med Open. 2022 Dec 13;8(1):147. doi: 10.1186/s40798-022-00542-8	Tsai MK	Sports Med Open	2022	2022/12/13	PMC9746582		10.1186/s40798-022-00542-8
25869658	Feasibility and safety of intradialysis yoga and education in maintenance hemodialysis patients	Birdee GS, Rothman RL, Sohl SJ, Wertenbaker D, Wheeler A, Bossart C, Balasire O, Ikizler TA	J Ren Nutr. 2015 Sep;25(5):445-53. doi: 10.1053/j.jrn.2015.02.004. Epub 2015 Apr 11	Birdee GS	J Ren Nutr	2015	2015/4/15	PMC4863695	NIHMS778142	10.1053/j.jrn.2015.02.004
41141526	Patient activation impacts exercise in older adults with kidney disease	Mirpuri KK, Roach C, Woodall EM, Prigmore HL, Greevy RA, Wilkinson T, Lightfoot C, Bonnet KR, Cukor D, Taylor WD, Bahri N, Umeukeje EM, Burdick R, Schlundt DG, Cavanaugh KL, Nair D	Kidney Int Rep. 2025 Jul 23;10(10):3431-3443. doi: 10.1016/j.ekir.2025.07.017. eCollection 2025 Oct	Mirpuri KK	Kidney Int Rep	2025	2025/10/27	PMC12545686		10.1016/j.ekir.2025.07.017
31161521	Attitudes of hemodialysis patients, medical and nursing staff towards patients' physical activity	Michou V, Kouidi E, Liakopoulos V, Dounousi E, Deligiannis A	Int Urol Nephrol. 2019 Jul;51(7):1249-1260. doi: 10.1007/s11255-019-02179-1. Epub 2019 Jun 3	Michou V	Int Urol Nephrol	2019	2019/6/5	-	-	10.1007/s11255-019-02179-1
40692323	Effects of reducing sedentary behaviour on renal glucose uptake during insulin stimulation: A post-hoc analysis of a 6-month randomized controlled trial	Rebelos E, Dadson P, Sjöros T, Laine S, Norha J, Garthwaite T, Löyttyniemi E, Eskola O, Koivumäki M, Vähä-Ypyä H, Sievänen H, Vasankari T, Hirvonen J, Laitinen K, Houttu N, Kalliokoski KK, Knuuti J, Ferrannini E, Mari A, Heinonen I	Diabetes Obes Metab. 2025 Oct;27(10):5772-5781. doi: 10.1111/dom.16631. Epub 2025 Jul 22	Rebelos E	Diabetes Obes Metab	2025	2025/7/22	PMC12409238		10.1111/dom.16631
21795245	Barriers to exercise participation among dialysis patients	Delgado C, Johansen KL	Nephrol Dial Transplant. 2012 Mar;27(3):1152-7. doi: 10.1093/ndt/gfr404. Epub 2011 Jul 26	Delgado C	Nephrol Dial Transplant	2012	2011/7/29	PMC3289894		10.1093/ndt/gfr404
29319765	Muscle thickness of the pectoralis major and rectus abdominis and level of physical activity in chronic hemodialysis patients	Bueno AF, Lemos FA, Ferrareze ME, Santos WAMD, Veronese FV, Dias AS	J Bras Nefrol. 2017 Oct-Dec;39(4):391-397. doi: 10.5935/0101-2800.20170071	Bueno AF	J Bras Nefrol	2017	2018/1/11	-	-	10.5935/0101-2800.20170071
24286579	Management of ACCF/AHA stage A and B patients	Subzposh F, Gupta A, Hankins SR, Eisen HJ	Cardiol Clin. 2014 Feb;32(1):63-71, viii. doi: 10.1016/j.ccl.2013.09.003	Subzposh F	Cardiol Clin	2014	2013/11/30	-	-	10.1016/j.ccl.2013.09.003
29947736	Prolonged leisure time spent sitting in relation to cause-specific mortality in a large US cohort	Patel AV, Maliniak ML, Rees-Punia E, Matthews CE, Gapstur SM	Am J Epidemiol. 2018 Oct 1;187(10):2151-2158. doi: 10.1093/aje/kwy125	Patel AV	Am J Epidemiol	2018	2018/6/28	PMC6454412		10.1093/aje/kwy125
24928604	Energy expenditure, spontaneous physical activity and with weight gain in kidney transplant recipients	Heng AE, Montaurier C, Cano N, Caillot N, Blot A, Meunier N, Pereira B, Marceau G, Sapin V, Jouve C, Boirie Y, Deteix P, Morio B	Clin Nutr. 2015 Jun;34(3):457-64. doi: 10.1016/j.clnu.2014.05.003. Epub 2014 May 15	Heng AE	Clin Nutr	2015	2014/6/15	-	-	10.1016/j.clnu.2014.05.003
25931456	Light-intensity physical activities and mortality in the United States general population and CKD subpopulation	Beddhu S, Wei G, Marcus RL, Chonchol M, Greene T	Clin J Am Soc Nephrol. 2015 Jul 7;10(7):1145-53. doi: 10.2215/CJN.08410814. Epub 2015 Apr 30	Beddhu S	Clin J Am Soc Nephrol	2015	2015/5/2	PMC4491291		10.2215/CJN.08410814
26864370	Dietary energy requirements in relatively healthy maintenance hemodialysis patients estimated from long-term metabolic studies	Shah A, Bross R, Shapiro BB, Morrison G, Kopple JD	Am J Clin Nutr. 2016 Mar;103(3):757-65. doi: 10.3945/ajcn.115.112995. Epub 2016 Feb 10	Shah A	Am J Clin Nutr	2016	2016/2/12	PMC4763489		10.3945/ajcn.115.112995
30596171	Health behaviors in younger and older adults with CKD: results from the CRIC study	Schrauben SJ, Hsu JY, Wright Nunes J, Fischer MJ, Srivastava A, Chen J, Charleston J, Steigerwalt S, Tan TC, Fink JC, Ricardo AC, Lash JP, Wolf M, Feldman HI, Anderson AH; CRIC Study Investigators	Kidney Int Rep. 2018 Sep 17;4(1):80-93. doi: 10.1016/j.ekir.2018.09.003. eCollection 2019 Jan	Schrauben SJ	Kidney Int Rep	2018	2019/1/1	PMC6308910		10.1016/j.ekir.2018.09.003
28181648	Prevalence and factors associated with faecal impaction in the Spanish old population	Serrano Falcón B, Álvarez Sánchez Á, Diaz-Rubio M, Rey E	Age Ageing. 2017 Jan 12;46(1):119-124. doi: 10.1093/ageing/afw166	Serrano Falcón B	Age Ageing	2017	2017/2/10	-	-	10.1093/ageing/afw166
37290375	Objective assessment of mobility among adults with diabetes and end-stage renal disease using walking aid: a cross-sectional cohort study	Mishra RK, Hamad A, Ibrahim R, Mathew M, Talal T, Al-Ali F, Park C, Davuluri V, Fernando ME, Najafi B	Clin Biomech (Bristol). 2023 Jul;107:106014. doi: 10.1016/j.clinbiomech.2023.106014. Epub 2023 May 25	Mishra RK	Clin Biomech (Bristol)	2023	2023/6/8	-	-	10.1016/j.clinbiomech.2023.106014
39519532	Integrated cardiorespiratory rehabilitation and its impact on cardio-renal-metabolic profile after cardiac surgery	Marek-Iannucci S, Palazzuoli A, Babarto M, Lazarevic Z, Beltrami M, Fedele F	Nutrients. 2024 Oct 30;16(21):3699. doi: 10.3390/nu16213699	Marek-Iannucci S	Nutrients	2024	2024/11/9	PMC11547743		10.3390/nu16213699
29346062	Television viewing time, physical activity, and mortality among African Americans	Imran TF, Ommerborn M, Clark C, Correa A, Dubbert P, Gaziano JM, Djoussé L	Prev Chronic Dis. 2018 Jan 18;15:E10. doi: 10.5888/pcd15.170247	Imran TF	Prev Chronic Dis	2018	2018/1/19	PMC5774305		10.5888/pcd15.170247
23479122	Physical activity and exercise training: a relevant aspect of the dialysis patient's care	Cupisti A, D'Alessandro C, Bottai A, Fumagalli G, Capitanini A	Intern Emerg Med. 2013 Apr;8 Suppl 1:S31-4. doi: 10.1007/s11739-013-0917-y	Cupisti A	Intern Emerg Med	2013	2013/3/13	-	-	10.1007/s11739-013-0917-y
33207339	Leisure-time physical activity and mortality in CKD: a 1999-2012 NHANES analysis	Zhang NH, Luo R, Cheng YC, Ge SW, Xu G	Am J Nephrol. 2020;51(11):919-929. doi: 10.1159/000511685. Epub 2020 Nov 18	Zhang NH	Am J Nephrol	2020	2020/11/18	-	-	10.1159/000511685
22717340	Association of sitting time and physical activity with CKD: a cross-sectional study in family practices	Bharakhada N, Yates T, Davies MJ, Wilmot EG, Edwardson C, Henson J, Webb D, Khunti K	Am J Kidney Dis. 2012 Oct;60(4):583-90. doi: 10.1053/j.ajkd.2012.04.024. Epub 2012 Jun 19	Bharakhada N	Am J Kidney Dis	2012	2012/6/22	-	-	10.1053/j.ajkd.2012.04.024
40111717	The relationship between physical inactivity and sexual dysfunction among patients receiving hemodialysis	Cruz-Bañares A, Rubio-Zapata HA, Estrella-Castillo D, Flores-Tapia JP, Gutiérrez-Mac GA	Int Urol Nephrol. 2025 Aug;57(8):2649-2656. doi: 10.1007/s11255-025-04460-y. Epub 2025 Mar 20	Cruz-Bañares A	Int Urol Nephrol	2025	2025/3/20	-	-	10.1007/s11255-025-04460-y
29211840	Occupational health risks and intervention strategies for US taxi drivers	Murray KE, Buul A, Aden R, Cavanaugh AM, Kidane L, Hussein M, Eastman A, Checkoway H	Health Promot Int. 2019 Apr 1;34(2):323-332. doi: 10.1093/heapro/dax082	Murray KE	Health Promot Int	2019	2017/12/7	PMC6445341		10.1093/heapro/dax082
25501981	Clinical determinants of reduced physical activity in hemodialysis and peritoneal dialysis patients	Cobo G, Gallar P, Gama-Axelsson T, Di Gioia C, Qureshi AR, Camacho R, Vigil A, Heimbürger O, Ortega O, Rodriguez I, Herrero JC, Bárány P, Lindholm B, Stenvinkel P, Carrero JJ	J Nephrol. 2015 Aug;28(4):503-10. doi: 10.1007/s40620-014-0164-y. Epub 2014 Dec 12	Cobo G	J Nephrol	2015	2014/12/16	-	-	10.1007/s40620-014-0164-y
30779818	Prevalence and associated factors of uncontrolled blood pressure among hypertensive patients in the rural communities in the central areas in Thailand: A cross-sectional study	Meelab S, Bunupuradah I, Suttiruang J, Sakulrojanawong S, Thongkua N, Chantawiboonchai C, Chirabandhu P, Lertthanaporn S, Suwanthip K, Songsaengthum C, Keattisaksri B, Trakulsuk P, Pittapun A, Nata N, Rangsin R, Sakboonyarat B	PLoS One. 2019 Feb 19;14(2):e0212572. doi: 10.1371/journal.pone.0212572. eCollection 2019	Meelab S	PLoS One	2019	2019/2/20	PMC6380583		10.1371/journal.pone.0212572
28558794	Assessment of physical activity, capacity and nutritional status in elderly peritoneal dialysis patients	Cupisti A, D'Alessandro C, Finato V, Del Corso C, Catania B, Caselli GM, Egidi MF	BMC Nephrol. 2017 May 30;18(1):180. doi: 10.1186/s12882-017-0593-7	Cupisti A	BMC Nephrol	2017	2017/6/1	PMC5450102		10.1186/s12882-017-0593-7
24762526	TV watching, but not physical activity, is associated with change in kidney function in older adults	Hawkins M, Newman AB, Madero M, Patel KV, Shlipak MG, Cooper J, Johansen KL, Navaneethan SD, Shorr RI, Simonsick EM, Fried LF	J Phys Act Health. 2015 Apr;12(4):561-8. doi: 10.1123/jpah.2013-0289. Epub 2014 Apr 17	Hawkins M	J Phys Act Health	2015	2014/4/26	PMC4816441	NIHMS602069	10.1123/jpah.2013-0289
33175177	Trends in poor health indicators among Black and Hispanic middle-aged and older adults in the United States, 1999-2018	Odlum M, Moise N, Kronish IM, Broadwell P, Alcántara C, Davis NJ, Cheung YKK, Perotte A, Yoon S	JAMA Netw Open. 2020 Nov 2;3(11):e2025134. doi: 10.1001/jamanetworkopen.2020.25134	Odlum M	JAMA Netw Open	2020	2020/11/11	PMC7658737		10.1001/jamanetworkopen.2020.25134
20811334	Low level of self-reported physical activity in ambulatory patients new to dialysis	Johansen KL, Chertow GM, Kutner NG, Dalrymple LS, Grimes BA, Kaysen GA	Kidney Int. 2010 Dec;78(11):1164-70. doi: 10.1038/ki.2010.312. Epub 2010 Sep 1	Johansen KL	Kidney Int	2010	2010/9/3	PMC4170106	NIHMS628523	10.1038/ki.2010.312
41180192	Metabolites mediate the effects of healthy lifestyles on the risks of common age-related diseases	Li Y, Li H, Chen X, Liang X	Front Endocrinol (Lausanne). 2025 Oct 17;16:1654172. doi: 10.3389/fendo.2025.1654172. eCollection 2025	Li Y	Front Endocrinol (Lausanne)	2025	2025/11/3	PMC12575140		10.3389/fendo.2025.1654172
36707111	Effects of intradialytic inspiratory muscle training at different intensities on diaphragm thickness and functional capacity: clinical trial protocol in patients undergoing haemodialysis	Teixeira MS, Ferrari F, Dipp T, Carvalho G, Bitencourt EDS, Saffi M, Stein R	BMJ Open. 2023 Jan 27;13(1):e066778. doi: 10.1136/bmjopen-2022-066778	Teixeira MS	BMJ Open	2023	2023/1/27	PMC9884932		10.1136/bmjopen-2022-066778
24964642	Physical activity in end-stage renal disease patients: a pilot project in Puerto Rico	Amaral-Figueroa MI	P R Health Sci J. 2014 Jun;33(2):74-9	Amaral-Figueroa MI	P R Health Sci J	2014	2014/6/27	-	-	
24656397	Physical function and physical activity assessment and promotion in the hemodialysis clinic: a qualitative study	Painter P, Clark L, Olausson J	Am J Kidney Dis. 2014 Sep;64(3):425-33. doi: 10.1053/j.ajkd.2014.01.433. Epub 2014 Mar 20	Painter P	Am J Kidney Dis	2014	2014/3/25	-	-	10.1053/j.ajkd.2014.01.433
40709207	Translation of clinical practice to research: the VETS and ETHOS epidemiologic prospective cohorts	Myers J, Kokkinos P, Samuel IBH, Faselis C, Fletcher R, Froelicher V	Front Cardiovasc Med. 2025 Jul 10;12:1577931. doi: 10.3389/fcvm.2025.1577931. eCollection 2025	Myers J	Front Cardiovasc Med	2025	2025/7/25	PMC12288094		10.3389/fcvm.2025.1577931
28882818	Prospective association of physical activity and heart failure hospitalizations among Black adults with normal ejection fraction: the Jackson heart study	Koo P, Gjelsvik A, Choudhary G, Wu WC, Wang W, McCool FD, Eaton CB	J Am Heart Assoc. 2017 Sep 7;6(9):e006107. doi: 10.1161/JAHA.117.006107	Koo P	J Am Heart Assoc	2017	2017/9/9	PMC5634276		10.1161/JAHA.117.006107
35911503	Mitigation of MAFLD in high fat-high sucrose-fructose fed mice by a combination of genistein consumption and exercise training	St Aubin CR, Fisher AL, Hernandez JA, Broderick TL, Al-Nakkash L	Diabetes Metab Syndr Obes. 2022 Jul 23;15:2157-2172. doi: 10.2147/DMSO.S358256. eCollection 2022	St Aubin CR	Diabetes Metab Syndr Obes	2022	2022/8/1	PMC9329575		10.2147/DMSO.S358256
23218530	Physical exercise related improvement in obstructive sleep apnea. Look for the rostral fluid shift	Mirrakhimov AE	Med Hypotheses. 2013 Feb;80(2):125-8. doi: 10.1016/j.mehy.2012.11.007. Epub 2012 Dec 3	Mirrakhimov AE	Med Hypotheses	2013	2012/12/11	-	-	10.1016/j.mehy.2012.11.007
22382216	Cardiovascular disease in patients with end-stage renal disease on hemodialysis in a developing country	Silva LS, Oliveira RA, Silva GB, Lima JW, Silva RP, Liborio AB, Daher EF, Sobrinho CR	Saudi J Kidney Dis Transpl. 2012 Mar;23(2):262-6	Silva LS	Saudi J Kidney Dis Transpl	2012	2012/3/3	-	-	-
40639457	Association of self-reported physical activity with sarcopenia in patients with kidney failure	Wong L, Schembri E, McMahon LP	Clin Nutr ESPEN. 2025 Oct;69:140-144. doi: 10.1016/j.clnesp.2025.07.007. Epub 2025 Jul 8	Wong L	Clin Nutr ESPEN	2025	2025/7/10	-	-	10.1016/j.clnesp.2025.07.007
34737308	The ability of physical activity in reducing mortality risks and cardiovascular loading and in extending life expectancy in patients with COPD	Shu CC, Lee JH, Tsai MK, Su TC, Wen CP	Sci Rep. 2021 Nov 4;11(1):21674. doi: 10.1038/s41598-021-00728-2	Shu CC	Sci Rep	2021	2021/11/5	PMC8569178		10.1038/s41598-021-00728-2
23541222	Impact of objectively measured sedentary behaviour on changes in insulin resistance and secretion over 3 years in the RISC study: interaction with weight gain	Lahjibi E, Heude B, Dekker JM, Højlund K, Laville M, Nolan J, Oppert JM, Balkau B; RISC Study Group	Diabetes Metab. 2013 May;39(3):217-25. doi: 10.1016/j.diabet.2012.12.006. Epub 2013 Mar 27	Lahjibi E	Diabetes Metab	2013	2013/4/2	-	-	10.1016/j.diabet.2012.12.006
31256507	Heart failure with preserved ejection fraction: a growing global epidemic	Naing P, Forrester D, Kangaharan N, Muthumala A, Mon Myint S, Playford D	Aust J Gen Pract. 2019 Jul;48(7):465-471. doi: 10.31128/AJGP-03-19-4873	Naing P	Aust J Gen Pract	2019	2019/7/1	-	-	10.31128/AJGP-03-19-4873
26765515	Quantifying physical activity levels and sleep in hemodialysis patients using a commercially available activity tracker	Han M, Williams S, Mendoza M, Ye X, Zhang H, Calice-Silva V, Thijssen S, Kotanko P, Meyring-Wösten A	Blood Purif. 2016;41(1-3):194-204. doi: 10.1159/000441314. Epub 2016 Jan 15	Han M	Blood Purif	2016	2016/1/15	-	-	10.1159/000441314
25185681	Determinants of health check attendance in adults: findings from the cross-sectional German Health Update (GEDA) study	Hoebel J, Starker A, Jordan S, Richter M, Lampert T	BMC Public Health. 2014 Sep 4;14:913. doi: 10.1186/1471-2458-14-913	Hoebel J	BMC Public Health	2014	2014/9/5	PMC4167266		10.1186/1471-2458-14-913
21329632	Assessment of habitual physical activity and energy expenditure in dialysis patients and relationships to nutritional parameters	Cupisti A, Capitanini A, Betti G, D'Alessandro C, Barsotti G	Clin Nephrol. 2011 Mar;75(3):218-25. doi: 10.5414/cnp75218	Cupisti A	Clin Nephrol	2011	2011/2/19	-	-	10.5414/cnp75218
24436478	Clinical correlates of insulin sensitivity and its association with mortality among men with CKD stages 3 and 4	Xu H, Huang X, Arnlöv J, Cederholm T, Stenvinkel P, Lindholm B, Risérus U, Carrero JJ	Clin J Am Soc Nephrol. 2014 Apr;9(4):690-7. doi: 10.2215/CJN.05230513. Epub 2014 Jan 16	Xu H	Clin J Am Soc Nephrol	2014	2014/1/18	PMC3974352		10.2215/CJN.05230513
25053714	Attenuating the mortality risk of high serum uric acid: the role of physical activity underused	Chen JH, Wen CP, Wu SB, Lan JL, Tsai MK, Tai YP, Lee JH, Hsu CC, Tsao CK, Wai JP, Chiang PH, Pan WH, Hsiung CA	Ann Rheum Dis. 2015 Nov;74(11):2034-42. doi: 10.1136/annrheumdis-2014-205312. Epub 2014 Jul 22	Chen JH	Ann Rheum Dis	2015	2014/7/24	-	-	10.1136/annrheumdis-2014-205312
25764298	Changes in physical activity among adults with diabetes: a longitudinal cohort study of inactive patients with type 2 diabetes who become physically active	Palakodeti S, Uratsu CS, Schmittdiel JA, Grant RW	Diabet Med. 2015 Aug;32(8):1051-7. doi: 10.1111/dme.12748. Epub 2015 Apr 10.	Palakodeti S	Diabet Med	2015	2015/3/13	PMC5008143		10.1111/dme.12748
25278548	Associations of self-reported physical activity types and levels with quality of life, depression symptoms, and mortality in hemodialysis patients: the DOPPS	Lopes AA, Lantz B, Morgenstern H, Wang M, Bieber BA, Gillespie BW, Li Y, Painter P, Jacobson SH, Rayner HC, Mapes DL, Vanholder RC, Hasegawa T, Robinson BM, Pisoni RL	Clin J Am Soc Nephrol. 2014 Oct 7;9(10):1702-12. doi: 10.2215/CJN.12371213. Epub 2014 Oct 2	Lopes AA	Clin J Am Soc Nephrol	2014	2014/10/4	PMC4186524		10.2215/CJN.12371213
23591985	Combined walking exercise and alkali therapy in patients with CKD4-5 regulates intramuscular free amino acid pools and ubiquitin E3 ligase expression	Watson EL, Kosmadakis GC, Smith AC, Viana JL, Brown JR, Molyneux K, Pawluczyk IZ, Mulheran M, Bishop NC, Shirreffs S, Maughan RJ, Owen PJ, John SG, McIntyre CW, Feehally J, Bevington A	Eur J Appl Physiol. 2013 Aug;113(8):2111-24. doi: 10.1007/s00421-013-2628-5. Epub 2013 Apr 17	Watson EL	Eur J Appl Physiol	2013	2013/4/18	-	-	10.1007/s00421-013-2628-5
28820539	A survey of non-communicable diseases and their risk factors among university employees: a single institutional study	Agaba EI, Akanbi MO, Agaba PA, Ocheke AN, Gimba ZM, Daniyam S, Okeke EN	Cardiovasc J Afr. 2017 Nov/Dec 23;28(6):377-384. doi: 10.5830/CVJA-2017-021. Epub 2017 Aug 15	Agaba EI	Cardiovasc J Afr	2017	2017/8/19	PMC5885043		10.5830/CVJA-2017-021
25083558	Factors influencing prognosis and functional outcome one year after a first-time stroke in a Caribbean population	Galanth S, Tressieres B, Lannuzel A, Foucan P, Alecu C	Arch Phys Med Rehabil. 2014 Nov;95(11):2134-9. doi: 10.1016/j.apmr.2014.07.394. Epub 2014 Jul 30	Galanth S	Arch Phys Med Rehabil	2014	2014/8/2	-	-	10.1016/j.apmr.2014.07.394
23686710	Exercise training normalizes the blunted central component of the baroreflex in rats with heart failure: role of the PVN	Patel KP, Salgado HC, Liu X, Zheng H	Am J Physiol Heart Circ Physiol. 2013 Jul 15;305(2):H173-81. doi: 10.1152/ajpheart.00009.2013. Epub 2013 May 17	Patel KP	Am J Physiol Heart Circ Physiol	2013	2013/5/21	PMC3726956		10.1152/ajpheart.00009.2013
22528585	Influence of comorbidities and inactivity on the long-term outcomes of coronary artery bypass graft surgery in a small number of men on chronic hemodialysis	Gabaldon D, Xhu Z, Pett SB, Sun Y, Agaba EI, Servilla KS, Murata GH, Tzamaloukas AH	Int Urol Nephrol. 2013 Feb;45(1):293-5. doi: 10.1007/s11255-012-0172-6. Epub 2012 Apr 15	Gabaldon D	Int Urol Nephrol	2013	2012/4/25	-	-	10.1007/s11255-012-0172-6
